# Knowledge, attitude and practice on diet and physical activity among mothers with young children in the Jhaukhel-Duwakot Health Demographic Surveillance Site, Nepal

**DOI:** 10.1371/journal.pone.0200329

**Published:** 2018-07-09

**Authors:** Natalia Oli, Abhinav Vaidya, Katja Pahkala, Gabriele Eiben, Alexandra Krettek

**Affiliations:** 1 Department of Internal Medicine and Clinical Nutrition, Institute of Medicine, Sahlgrenska Academy at University of Gothenburg, Gothenburg, Sweden; 2 Department of Community Medicine, Kathmandu Medical College, Kathmandu, Nepal; 3 Paavo Nurmi Centre, Department of Health and Physical Activity, University of Turku, Turku, Finland; 4 Research Centre of Applied and Preventive Cardiovascular Medicine, University of Turku, Turku, Finland; 5 Department of Public Health and Community Medicine, University of Gothenburg, Gothenburg, Sweden; 6 Department of Biomedicine and Public Health, School of Health and Education, University of Skövde, Skövde, Sweden; 7 Department of Community Medicine, Faculty of Health Sciences, UiT The Arctic University of Norway, Tromsø, Norway; Public Library of Science, UNITED KINGDOM

## Abstract

The prevalence of cardiovascular diseases is increasing in low and middle-income countries; Nepal’s population shows a high prevalence of behavioral risk factors. Our cross-sectional study in the Jhaukhel-Duwakot Health Demographic Surveillance Site (JD-HDSS), located near the capital Kathmandu, explored knowledge, attitude, and practice (KAP) of mothers with young children regarding diet and physical activity and mothers’ perception of their children’s attitude and behavior toward the same issues. The purpose of our study was to assess needs of the mothers concerning cardiovascular health in general and more specifically regarding diet and physical activity, and to establish a baseline for future intervention in the community by comparing two villages of JD-HDSS. In August–November 2014, nine trained enumerators interviewed all mothers of children aged 1–7 years (N = 962). We scored responses on dietary and physical activity KAP, then categorized the scores based on the percentage obtained out of the maximum possible scores into “poor,” “fair,” and “good.” More highly educated mothers scored higher for KAP (all *p*<0.001); the children’s behavior score reflected their mother’s education level (*p* = 0.007). Most respondents were unfamiliar with the concept of healthy and unhealthy food. Overall, 57% of respondents in JD-HDSS had “good” knowledge, 44.6% had “good” attitude, and most (90%) had “poor” practice. We observed no significant differences between the villages regarding mothers’ knowledge and attitude or children’s behavior. Practice score of mothers in Jhaukhel was higher than those in Duwakot regarding diet and physical activity (*p*<0.001). Mothers’ perceived barriers for improving lifestyle were high cost of healthy food, taste preference of other family members, and lack of knowledge regarding healthy food. Barriers for physical activity were lack of leisure time, absence of parks and playgrounds, busy caring for children and old people, feeling lazy, and embarrassed to be physically active in front of others. Our findings suggest that a health education intervention promoting a healthy lifestyle for mothers and children might improve KAP and also improve cardiovascular health. To address mothers’ gap between knowledge and practice, a future intervention should consider perceived barriers.

## Introduction

Noncommunicable diseases (NCDs), particularly cardiovascular diseases (CVDs), are the leading cause of mortality and morbidity worldwide. Alarmingly, their prevalence is increasing drastically in low- and middle-income countries[1]. In Nepal, a low-income country, the high burden of NCDs account for 60% of all deaths; half of them contributed to CVDs[2].Nepal’s high prevalence of behavioral risk factors contributes to theincreasingfrequency of CVDs [3].

To address this issue, in 2010 we established a Health Demographic Surveillance Site (HDSS) in two neighboring villages, Duwakot and Jhaukhel, in the Bhaktapur district near Nepal’s capital city Kathmandu. Studies conducted in the Jhaukhel-Duwakot HDSS (JD-HDSS) demonstrated a high prevalence of CVD risk factors in both communities [4–6]. Low physical activity and unhealthy diet are common in JD-HDSS: more than one-third of respondents are inactive [4] and most do not consume five servings of fruits and vegetables per day, as recommended by the World Health Organization [6]. Similar to other low-income countries, Nepal faces a nutritional transition as it shifts from a traditional diet (i.e., high fiber, vegetables, and low-fat) to the western high-energy dense diet. This adoption of a western diet [7] is accompanied by a shift towards sedentary work and leisure time[8, 9]. This development is troublesome, as it is well established thatdiet and physical activity contribute to the etiology of CVDs and lifestyle modification is an important cornerstone for CVD prevention [10].

CVDs are rooted in childhood—mothers’ health status and environment during pregnancy and children’s environment after birth influence cardiovascular health in adulthood [11, 12].Several factors affect the early-life environment; e.g.,a family’s food environment is formed by parental knowledge about nutrition, parents’ feeding practices and cooking skills, accessibility and availability of food, and children’s individual characteristics [13, 14].Good nutritional knowledge of parents and parental support for physical activity associate with a healthier lifestyle in children [15]. In Nepal, mothers are primarily responsible for creating their children’s environment and lifestyle, which may affect children throughout life [16, 17]. Hence, directing lifestyle interventions towards mothers as key persons in the Nepalese context may help improve their children’s lifestyle. Subsequently, mothers may thereby contribute to the primordial prevention of NCDs.

We recently conducted focus group discussions to explore mothers’ perceptions of their children’s diet and physical activity in the JD-HDSS [18]. Irrespective of mothers’ educational level, all participants showed low health literacy regarding diet and physical activity. Moreover, participants believed that it was impossible to control their children’s eating habits and screen time [18]. Given these results, we undertook the current study to investigate in detail the overall knowledge, attitude, and practice (KAP) of mothers with young children regarding diet and physical activity, and mothers’ perception regarding their children’s attitude and behavior toward diet and physical activity. We also aimed to compare mothers’ KAP and children’s behavior between Duwakot and Jhaukhel. Such needs assessment identifies potential gaps in mothers’ KAP and children’s behavior and is crucial for planning a future community intervention.

## Materials and methods

### Study setting

We designed a cross-sectional analytical study to explore and compare JD-HDSS respondents’ KAP on diet and physical activity, and mothers’ perception regarding their young children’s attitude and behavior toward diet and physical activity. JD-HDSS is located near Nepal’s capital city Kathmandu. It comprises the two neighboring and urbanizing villages Duwakot and Jhaukhelthat havesimilar geo-ecological, ethnic, and cultural characteristics [19]. The villages have a combined population of 16,918 people (8,516 male and 8,402 female) in 3,505 households [19, 20].

### Study participants

From the JD-HDSS 2012 database, we created a list of all mothers with at least one child aged 1–7 years and intended to enroll all of these mothers in to our study. Mothers with hearing or mental disorders as well as mothers with mentally ill children or children whose health condition required special diet and physical regime were excluded from the study. All eligible mothers who were willing to participate were included in the study.

We identified and contacted 1,062 eligible mothers in the study area. The response rate was 93.5% in Duwakot and 91.8% in Jhaukhel. Altogether, 962 mothers (90.6%) completed forms that were used for analysis. Among them, 904 (94%) reported their monthly household income.

### Tools

We developed a structured questionnaire based on previous publications [21–23]. We tailored the questionnaire to the local context and discussed within our research group. We included questions on respondents’ demographic characteristics (e.g., age, education, occupation, type of family, etc.) as well as the occupation and education status of their husbands. This section also contained questions about children (e.g., age, sex, school grade, and primary caretaker). The main questionnaire contained three sections that focused on KAP. Each section included questions about diet and physical activity. Based on the Global Physical Activity Questionnaire (GPAQ), we described physical activity for respondents during work (including housework), travel to and from places, and leisure time [24]. Pictures with examples of common physical activities in the community were shown to mothers to further clarify the questions.

The *knowledge* section covered respondents’ knowledge of healthy and unhealthy diets and physical activity and their effects on cardiovascular health. Questions aimed to explore knowledge of healthy diet (e.g.,“What do you consider to be a healthy dietary habit for you?”) in a multiple response format with options such as “eating hygienic food irrespective of content,”“eating less sugar/sugary foods,” “eating smaller portions but more frequently,” “using less fat in cooking,” and “consuming more fruits and vegetables.” Furthermore, the questions provided some examples of food items (fresh salad, deep-fried vegetables, sweetened soft drinks, fruits, chips, etc.) and asked respondents to classify them as healthy or unhealthy. The questionnaire also mentioned different diseases (e.g., leprosy, heart diseases, malaria, diabetes, HIV/AIDS, etc.) and asked mothers if they associated development of these conditions with consumption of unhealthy food. The knowledge section also contained open-ended question (e.g., “What is junk food?”). We mentioned food components (i.e., sugar, salt, grains, fat, and fruits and vegetables) and asked respondents to define the amount (high, low, or not important) appropriate to a healthy diet. The section about knowledge of physical activity asked an open-ended question: “What is the role of physical activity for our health?” Another question included different statements (e.g.,“physical activity is bad for the heart, improves mental health, causes depression and frustration,” etc.) and asked respondents to either agree or disagree with the statements.

Questions in the *attitude* section explored mothers’ attitude toward their own and their children’s diet and physical activity. In addition, we examined mothers’ perception of their children’s attitude toward food and physical activity. We used 5-point Likert scale for most of the attitude questions. We provided different statements on diet and physical activity and asked mothers to choose between “strongly agree,” “agree,” “neutral”, “disagree,” “strongly disagree”. The food questions included statements such as “healthy food is not tasty;” “healthy food is enjoyable;” “healthy food is for sick people;” “if someone does enough exercise, can he/she eat whatever they like;” “it is good to buy soft drinks for the children because they are healthy;” “if you love your child, you should please him/her by buying favorite sweets,” etc. We also used “yes/no” questions when mentioned specific food items and asked respondents’ for their opinion about the taste of those foods. Additionally, we asked respondents’ perception of their own knowledge about healthy diet and physical activity and their ability to identify their main sources for health-related information. Examples of questions regarding respondents’ attitude toward their children’s diet and physical activity include: “What do you think affects your child’s food choice?” “Do you want to change your child's food pattern? If yes, how?” (open-ended question). “How would you rate your child’s physical activity status?” We also asked respondents about perceived barriers that prevent them and their children from eating a more healthy diet and being more physically active (“yes/no” questions).

Similarly, questions related to *practice* explored mothers’ own practice and practice toward their children regarding diet and physical activity. We also asked how respondents perceived their children’s dietary and physical activity behavior. Questions explored the availability of junk food and soft drinks at respondents’ home, types of food given to children at home, and mothers’ reaction when children demand junk food while shopping. We adopted the section about mothers’ physical activity practice from the GPAQ [24]. This section aimed to explore how much time per day and how many days per week mothers performed vigorous activities at work (e.g., lifting heavy loads) and during leisure time (e.g., running or other intensive sports activities). Likewise, questions explored mothers’ moderate physical activity at work (e.g., carrying light loads, housework) and leisure time (e.g., dancing). Travel time questions explored if mothers walked or cycled continuously for at least 10 minutes to reach work, market, etc.

Questions about children’s diet-related behavior aimed to inquire the mothers on how their child spend pocket money, the type of food children consume as snacks and during main meals, favorite food items and drinks, how often children consume soft drinks or prepackaged juices, and children’s eating habits while watching television. Questions that explored children’s physical activity included: “How long does the child walk to school?” “How long does he/she spend time doing homework?” “Does he/she spend time playing outside?” We also asked questions about children’s screen time.

To validate our questionnaire, we translated it from English into Nepali language and back-translated to English. We did pre-testing of the questionnaire in Changunarayan, a neighboring and socio-culturally similar village. Our pre-testing included 85 mothers with children aged 1–7 years, which is about 9% of the total number of eligible mothers in the study site [25]. Based on the pretesting results, we made necessary changes in the questionnaire to make it clearer for the respondents. Furthermore, we also checked the questionnaire for internal consistency (Cronbach’s alpha = 0.7).

### Data collection

Our criteria for recruitment of enumerators for data collection were females from the local communities with education level completed grade 10 and previous experience in data collection. We interviewed the applicants, and in deference to the traditional and cultural aspects of Nepalese society, recruited nine female enumerators. To supervise the data collection process, we recruited three Bachelor of Public Health graduates with previous experience in field work. NO and AV conducted 3-day training for the field supervisors. NO, AV and field supervisors conducted 6 days training (5 hours each day) on data collection for the enumerators. The enumerators conducted door-to-door visits to all of the listed mothers between September-November 2014 and interviewed eligible mothers, using the questionnaire. In households containing more than one eligible mother, enumerators applied a lottery method to select one interviewee. Enumerators contacted mothers who were not at home during the household visit by phone and met them later according to the mothers’ convenience.

### Data analysis

Trained operators initially entered data into Epidata 3.1 software. Next, we transferred and analyzed the data using SPSS, version 22.0. We scored knowledge questions as 1 and 0 for correct and incorrect answers, respectively. For example, for the multiple option question “What do you consider to be a healthy dietary habit for you?”, the ‘yes’ response to the option“eating hygienic food irrespective of content” scored 0, whereas the ‘yes’ response to the option “eating less sugar/sugary foods” scored 1. Furthermore, if the respondent considered chips, deep-fried vegetables, sugar-sweetened carbonated drinks as healthy food, she was given 0 scores for each of these food items and 1 score if they thought of themto be unhealthy. In case of open ended questions such as “What is junk food?”, we gave score 1 for correct answer and 0 for incorrect or for “don’t know” answer.

We scored attitude questions from 1to 5 (from strongly disagree to strongly agree),giving maximum scores for a positive attitude about healthy diet and physical activity [26]. In the practice section related to mothers’ physical activity,we converted responses to metabolic equivalent to task (MET)–minutes/week according to the GPAQ Analysis Guide [24]. We further categorized mothers’ physical activity as high, moderate,or low depending on their total MET–minutes/week. Mothers with high physical activity received 2 points, compared to 1 point for moderate physical activity and 0 points for mothers with low physical activity. Furthermore, mothers who were sedentary for 3 hours per day received 0 points, compared to 1 point for mothers who sat less than 3 hours per day.

We also scored children’s behavior. In families with more than one child aged 1–7, we selected the oldest child for data analysis. For example, questions about food items children usually ate hadthe options of “often”, “sometimes, “seldom,” and “never”. Answer “often” for healthy food such as egg, milk, rice and vegetables was given score 3, whereas “sometimes”, “seldom” and “never” scored 2, 1 and 0, respectively. In case of unhealthy food items such as sweets, instant noodles, chips, scores 0, 1, 2 and 3 were given for “often”, sometimes”, “seldom” and “never” respectively. Children with positiveor negative dietary and physical behavior received 1 or 0 points, respectively. Multiple option questions with yes/no answer such as “What is your child’ favorite drink?” were given 1 score for healthy items such as water, fresh juice. Children who prefer sugar-sweetened carbonated drinks, packed juice got 0 score. Children who played outside more than 3 hours per day received 5 scores, while those who played for 2–3 hours, 1–2 hours, 30 min- 1 hour, less than 30 minutes, and who are not playing scored 4, 3, 2, 1and 0, respectively. Similarly, screening time was scored: lowest score (i.e. 0) for more than 3 hours per day and highest score (i.e. 5) for those who spend less than 30 minutes.

We combined relevant scores to calculate four composite scores: three for mothers’ KAP andone for children’s behavior. Furthermore, we categorized mothers’ KAP scores into three categories based on the percentage of the maximum possible scores: “poor” (0%-50%), “fair” (51%-75%), or “good” (76%-100%). Similarly, we categorized children’s behavior scores into “poor”, “fair” or good” categories. The maximum possible scores for mothers’ knowledge, attitude, and practice were 73, 192, and 17, respectively. The maximum score for children’s behavior was 110. We performed Kruskal–Wallis test to determine the relationship between respondents’ KAP and demographic variables such as age, religion, ethnicity, average monthly household income, mothers’ education, and occupation. We applied the same test to investigate a possible association of children’s behavior scores with mothers’ level of education, occupation and household income.

All data were checked for normal distribution using Shapiro-Wilk test. Since data were not-normally distributed, we calculated median and interquartile range (IQR) for KAPscores regarding diet and physical activity. KAP scores from Duwakot and Jhaukhel villageswere compared using Mann–Whitney U test. We also applied Chi- squared test to compare categories of KAP scores between the villages. *P*<0.05 was considered significant.

### Ethical consideration

The Nepal Health Research Council granted ethical permission for this study (No. 150/2014). We also consulted with local leaders and health and administrative authorities in both villages, who granted their approval. Trained enumerators solicited informed verbal consent from the eligible mothers. This was done as many mothers in our study were illiterate and were therefore unable to provide written consent. More importantly, even literate respondents hesitated signing documents because of cultural inhibitions, which if challenged, could be even counter-productive for their participation. All respondents were informed that they were free to leave the study at any time. Privacy and confidentiality was maintained during all interviews. No external observers were present during data collection. Filled questionnaires were kept in the JD-HDSS office and all data were accessible only to the research team.

## Results

### Demographic characteristics

Table 1 shows the demographic characteristics of respondents (673 mothers with children aged 1–7 years in Duwakot and 289 in Jhaukhel). Respondents’ median age (IQR) 29 (6) and 28 (6) years, respectively, and their age distribution, educational status, and religion of the respondents were similar in both villages. However, the villages differed regarding mothers’ ethnicity, occupation, average monthly income of the household, and type of family (*p*<0.05). One-quarter of the mothers (22.8%) had not completed primary school (<5 grades). Although Newar, Hill Brahmins, and Chhetri were the prevalent ethnic groups in both communities, Chhetris were more common (35.4%) in Duwakot and Newars were more common (39.8%) in Jhaukhel. Almost 60% of the respondents in Duwakot lived in nuclear families, whereas about 60% of mothers in Jhaukhel lived in extended families. Most respondents (73.3%) in both villages were housewives. Furthermore, the monthly householdincome of most respondents (71.7%) was < 20,000 Nepalese rupees (NPR). More households in Jhaukhel than Duwakot (32.7% vs. 23%, respectively) had a monthly income of < 10,000 NPR. In most families (89%), the mother was the primary caregiver of their children.

**Table 1:** Comparison of Duwakot and Jhaukhel villages according to demographic variables.

Variables	Duwakot, N (%)	Jhaukhel, N (%)	Total, N (%)	*P* value ^a^
**Age (years)**				
19–25	161 (23.9)	62 (21.5)	223 (23.2)	0.681
26–35	459 (68.2)	205 (70.9)	664 (69.0)	
36–48	53 (7.9)	22 (7.6)	75 (7.8)	
**Education**				
<5 grade	163 (22.4)	56 (19.4)	219 (22.8)	
5–10 grade	330 (49)	135 (46.7)	465 (48.3)	0.052
>10 grade	180 (26.7)	98 (33.9)	278 (28.9)	
**Religion**				
Hindu	615 (91.4)	268 (92.7)	883 (91.8)	
Buddhism	33 (4.9)	10 (3.5)	43 (4.5)	0.611
Others^b^	25 (3.7)	11 (3.8)	36 (3.7)	
**Ethnicity**				
Newar	157 (23.3)	115 (39.8)	272 (28.3)	
Hill Brahmins	136 (20.2)	86 (29.8)	222 (23.1)	
Chhetri	238 (35.4)	41 (14.2)	279 (29)	<0.001
Hill ethnic caste^c^	73 (10.8)	31 (10.7)	104 (10.8)	
Others^d^	69 (10.3)	16 (5.5)	85 (8.8)	
**Family structure**				
Nuclear	398 (59.1)	121 (41.9)	519 (54.0)	<0.001
Extended	275 (40.9)	168 (58.1)	443 (46.0)	
**Mothers’ main occupation**				
Agriculture	28 (4.2)	3 (1.0)	31 (3.2)	
Office	23 (3.4)	9 (3.1)	32 (3.3)	
Labor	50 (7.4)	15 (5.2)	65 (6.8)	0.045
Self-employed	83 (12.3)	46 (15.9)	129 (13.4)	
Housewife	489 (72.7)	216 (74.7)	705 (73.3)	
**Average monthly household income (NPR)^e^^,^^f^**				
<10,000	146 (23)	88 (32.7)	234 (25.9)	
10,000–19,999	301 (47.4)	113 (42)	414 (45.8)	
20,000–29,999	93 (14.6)	42 (15.6)	135 (14.9)	0.016
30,000–39,999	42 (6.6)	11 (4.1)	53 (5.9)	
>40,000	53 (8.3)	15 (5.6)	68 (7.9)	
**Total**	**673**	**289**	**962**	

Classification of ethnic groups is based on the National Central Bureau of Statistics [27].

^a^ Obtained from a χ^2^ test.

^b^Other religion includes Christianity and Islam.

^c^Hill ethnic castes include Tamang, Dalit, Thakuri, Magar, and Rai.

^d^Other ethnicity includes Lama, Sherpa, Madeshi, Gurung, Tharu.

^e^NRs = Nepalese rupees (1 USD = NRs 106, approximately)

^f^ Total number of respondents for average monthly household income = 904 (635 in Duwakot and 269 in Jhaukhel)

### Mothers’ KAP scores

When we categorized respondents’ scores into “poor,” “fair,” and “good” groups, we found that 57% had good knowledge, 45% had good attitude, and most (90%) had poor practice.

**Fig 1:**
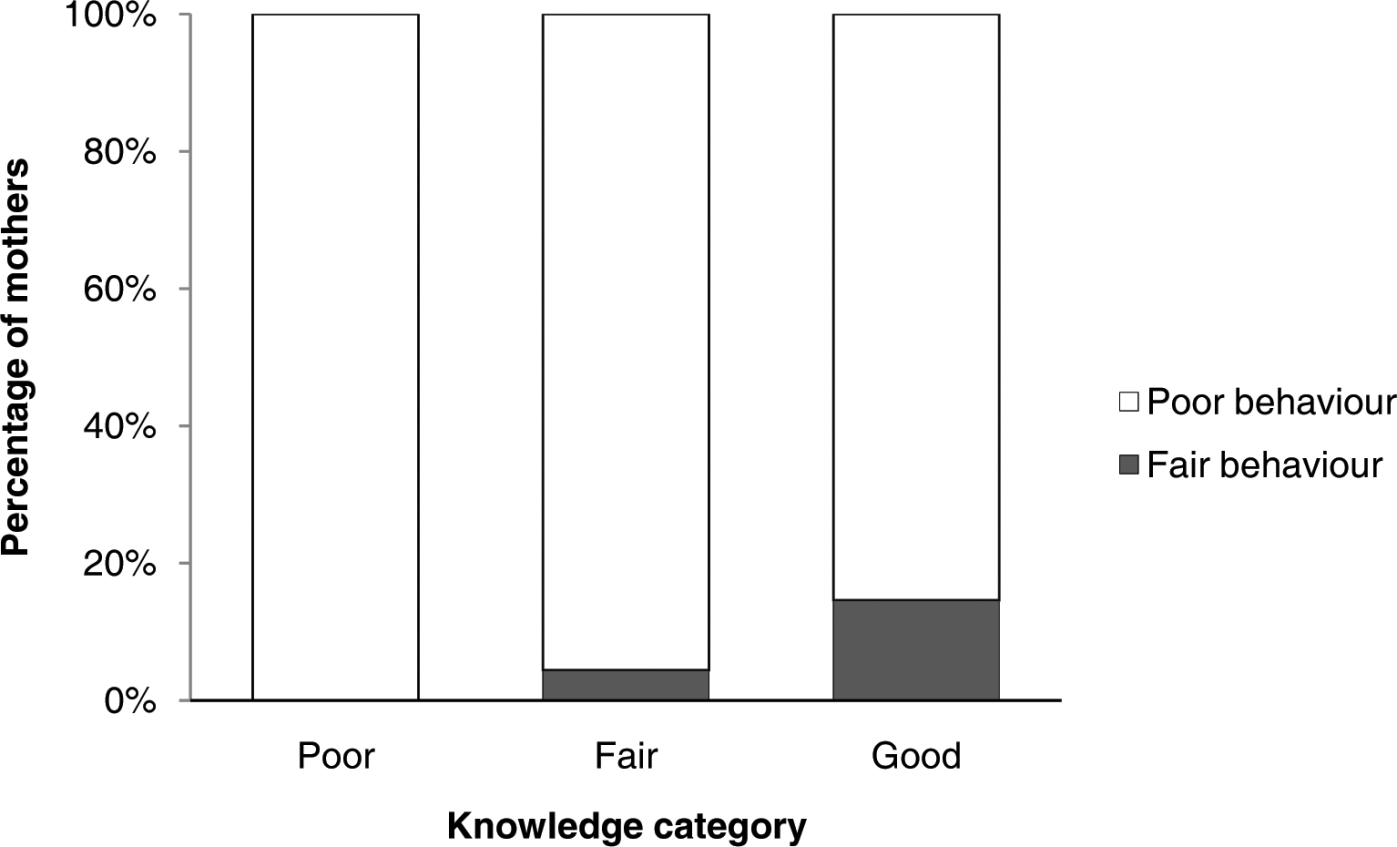
Levels of mothers’ practice score according to their knowledge score categories. KAP scores were categorized into three categories based on the percentage of the maximum possible scores: “poor” (0%-50%), “fair” (51%-75%), or “good” (76%-100%).

We further analyzed the distribution of mothers’ practice according to their knowledge categories (Fig 1). All mothers fell into either “poor” or “fair” categories (i.e., no one was in the “good” category). The percentage of mothers with fair practice was higher among mothers with “good” knowledge (14.6%), compared to mothers with “fair” (4.4%) or “poor” knowledge (0.0%).

### Association of mothers’ KAP scores with demographic variables

**Fig 2:**
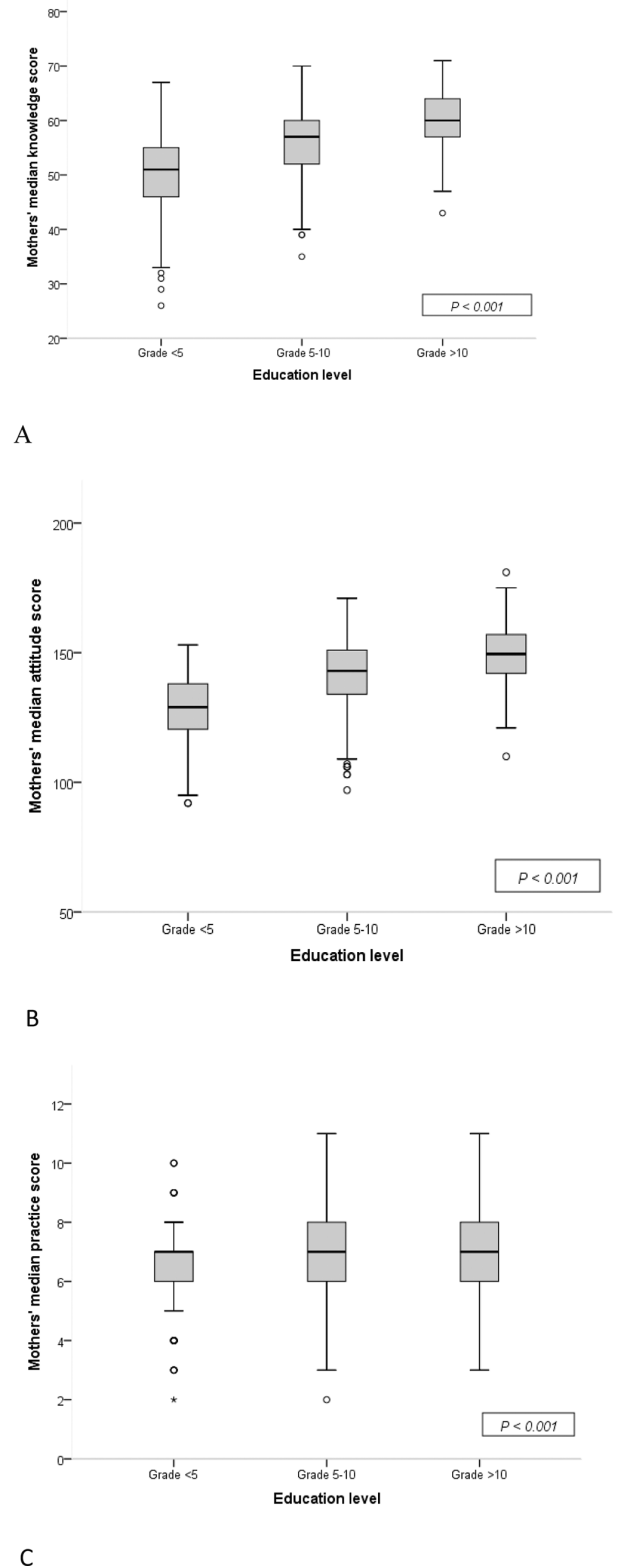
Mothers’ knowledge (A), attitude (B) and practice scores (C) according to their education level (N = 962).

Mothers aged26-35 years had higher median knowledge (*p*<0.001), attitude (*p* = 0.037), and practice (*p*<0.001) scores compared to younger or older women. KAP scores regarding diet and physical activity increased with level of education (Fig 2).

Respondents’ knowledge and attitude scores increased up to a household income of NPR 20,000–29,999 per month (*p*<0.001) and leveled off thereafter. Mothers’ practice scores did not change with increasing household income (*p*<0.14).

We also examined whether respondents’ KAP scores linked with their religion and ethnic origin. Hill Brahmin women had higher KAP scores regarding diet and physical activity, followed by Chhetris and Newars (*p*<0.001). Furthermore, Hindu mothers scored higher than Buddhist mothers and other religions (*p*<0.001). Additionally, mothers who worked as laborers or in the agricultural sector had lower knowledge and attitude scores compared to other occupations (*p*<0.001). Mothers who worked in offices or were self-employed scored higher onpractice than mothers who were housewives or who worked as laborers or in the agricultural sector (*p*<0.001). Regarding family structure, mothers in extended families had higher KAP scores than those in nuclear families (*p*<0.001 for knowledge, *p*<0.001 for attitude, and *p* = 0.033 for practice).

### Comparison of mothers’ KAP between the villages of JD-HDSS

Median (IQR) scores for mothers’ knowledge were 57 (10) in Duwakot and 57 (9) in Jhaukhel (*p* = 0.771). Median (IQR) scores for attitude were 142 (20) in Duwakot and 143 (18) in Jhaukhel (*p* = 0.588). Furthermore, mothers’ practice scores were 7 (2) and 7 (2) in Duwakot and Jhaukhel, respectively (*p*<0.001).

We observed no significant differences between Duwakot and Jhaukhel in the categories for mothers’ knowledge and attitude and children’s behavior (Table 2). However, more mothers in Duwakot had “poor” practice compared to those in Jhaukhel (*p* = 0.032).

**Table 2:** Comparison of knowledge, attitude, and practice scores categories regarding diet and physical activity between Duwakot and Jhaukhel.

Categories	Mothers						Children	
	Knowledge (N, %)		Attitude (N, %)		Practice* (N, %)		Behavior (N, %)	
	Duwakot	Jhaukhel	Duwakot	Jhaukhel	Duwakot	Jhaukhel	Duwakot	Jhaukhel
Poor	8 (1.2)	0 (0.0)	2 (0.3)	1 (0.3)	613 (91.1)	251 (86.9)	1 (0.1)	0 (0.0)
Fair	285 (42.3)	121 (41.9)	372 (55.3)	159 (55.0)	60 (8.9)	38 (13.1)	669 (99.4)	288 (99.7)
Good	380 (56.5)	168 (58.1)	299 (44.4)	129 (44.6)	0 (0.0)	0 (0.0)	3 (0.4)	1 (0.3)

P value is significant (0.032)

### Mothers’ perceived barriers and supportive factors for healthy diet and physical activity

Respondents indicated that the most common barriers for healthy diet included high cost of healthy food (72%), difficulty giving up favorite food (70%), taste preference of other family members (69%), lack of knowledge regarding healthy food (68%), busy lifestyle (63%), and lack of cooking skills for healthy food (61%). Similarly, barriers for increased physical activity were lack of leisure time (84%), having to care for children or old people (82%), feeling lazy (79%), absence of parks and playgrounds (76%), and embarrassed to do physical activity in front of others (66%).

Factors that mothers perceived as improving their owndiet and physical activity included better information about healthy food (92%), importance of physical activity (97%), dietary advice from medical personnel (92%), and advice about physical activity (94%). Moreover, mothers also emphasized the importance of support from relatives and friends for healthy diet (87%) and physical activity (93%). They also thought that availability of playgrounds (93%), their own ill health (83%), and healthy diet and physical activity (55%) can facilitate positive behavior changes.

### Children’s behavior scores and their variations

The children in our study included 444 girls (46.2%) and 518 boys (53.8%). Median (IQR) score for children’s behavior, as perceived by their mothers, was 72 (6). The maximum possible score for children’s behavior regarding diet and physical activity was 110. Only four children (0.4%) had “good” behavior, while almost all of the children (99.5%) had “fair” behavior and one child (0.1%) had “poor” behavior for diet and physical activity. We did not assess the children’s knowledge and attitude about diet and physical activity.

### Association of children’s behavior scores with mothers’ demographic variables

**Fig 3:**
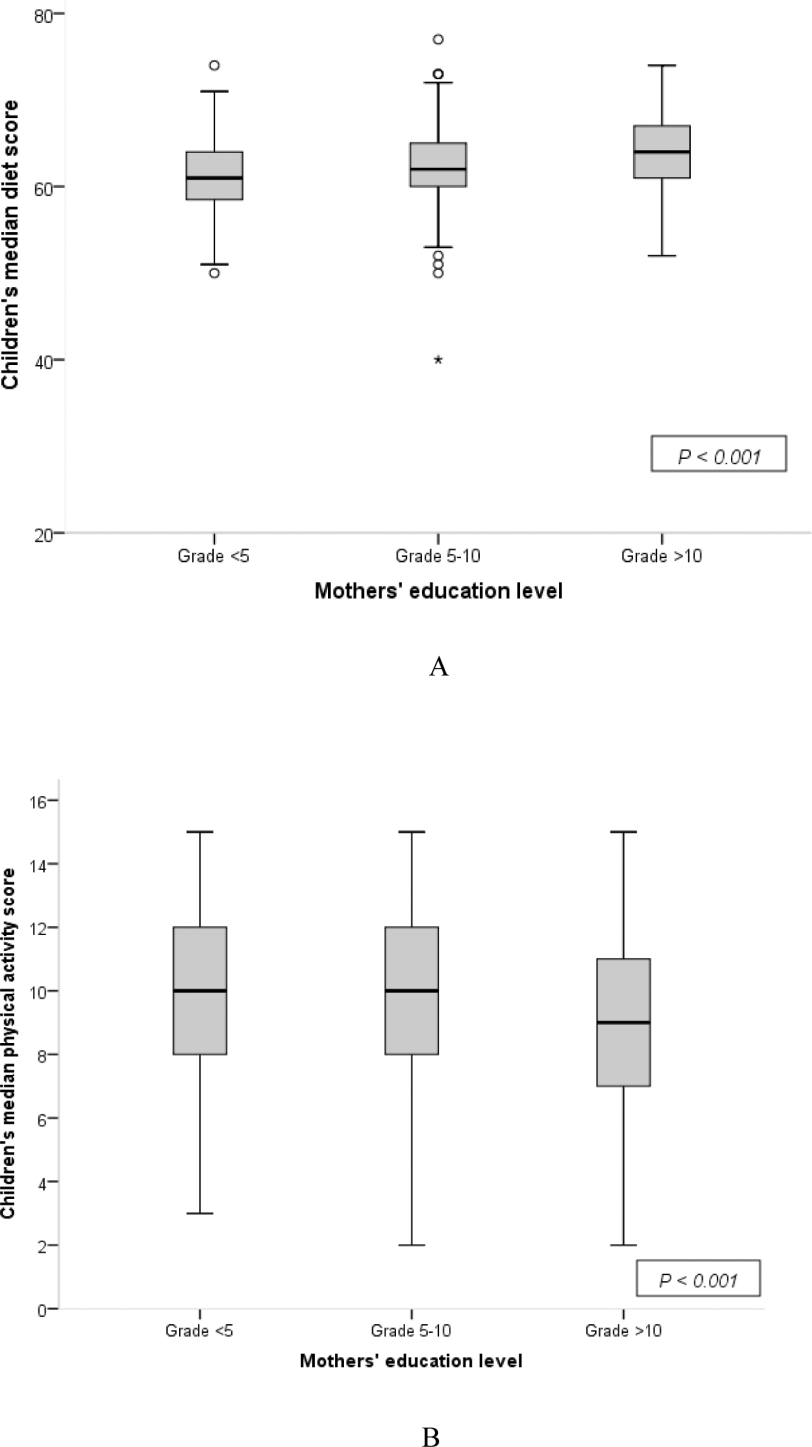
Children’s behavior scores on diet (A) and physical activity (B) according to their mothers’ education level (N = 962).

The children’s diet scores increased (*p*<0.001), whereas their physical activity scores decreased (*p*<0.001) with increase in their mothers’ level of education (Fig 3). On the other hand, monthly household income and mothers’ occupation (*p* = 0.41 and *p* = 0.39, respectively) did not associate with children’s behavior.

Similar to the mothers’ KAP scores, children’s behavior scores were higher among Hill Brahmins, followed by Chhetris and Newars (*p*<0.001). Children of Hindu mothers had higher behavior scores, followed by children of mothers with other religions (*p* = 0.001). Likewise, children’s behavior scores were higher in extended families than in nuclear families (p = 0.042), and their behavior scores were similar in mothers of different age groups (*p* = 0.943).

## Discussion

Until now, studies exploring mothers’ KAP regarding heart-healthy diet and physical activity and their association with children’s behavior have been done mostly in high-income countries[28, 29].To our knowledge, this is the first study conducted in a Nepalese population that focused on mothers’ KAP regarding diet and physical activity and their association with perceived habits in children. Our previous study in the adult population of JD-HDSS determineda high prevalence of cardiovascular risk factors and poor knowledge about the causes and symptoms of heart/cardiovascular disease [5]. The current study’s focus on mothers is important for the community andbridges the gap in the existing data.

### Mothers’ KAP variation according to sociodemographic characteristics

We found that though half of the mothers had “good” knowledge and attitude, most (90%) of them had “poor” practice. On the other hand, mothers with “good” knowledge showed better practice in diet and physical activity which is reflected also in higher KAP scores with increasing level of education. Studies among Omani and Dutch mothers support this finding [30, 31].

The fact that less-educated mothers who work as laborers or in agriculture scored lower on knowledge and attitude compared to mothers in other occupations, underpins the effect of education on KAP and its relation to diet and physical activity. Moreover, Hill Brahmins, Chhetri and Newar mothers, who traditionally have more access to education in Nepal, scored higher than other respondents [32]. Such association of KAP scores with education can be a good point to consider for health education intervention in the community to promote healthy lifestyle. Although higher income showed a positive effect on knowledge and attitude, it did not affect practice, possibly because mothers with higher income are more educated and have better access to different sources of information that may influence their knowledge and attitude on diet and physical activity. However, low motivation and low self- perceived control reported by the JD-HDSS mothers in our previous study may affect the likelihood of changing their behavior in spite of having “good” knowledge and attitude [18]. Low personal control can lead to apathy that makes it less likely that they can attain behavioral changes. Thus, people have a positive sense of perceived control when they believe that they control their own behavior (internal locus of control) and can actually make those changes (self-efficacy) [33, 34]. Indeed, maternal self-efficacy affects healthy behavior in women and their children, particularly their physical activity [35]. Hence, community interventions should also enhance mothers’ self–efficacy and low perceived control in order to improve their knowledge and attitude and, more importantly, their practice.

### Comparison of KAP of mothers in JD-HDSS villages

Our comparison of mothers’ knowledge and attitude between Duwakot and Jhaukhel revealed no significant differences, although mothers in Jhaukhel had better practice than those in Duwakot, possibly because more mothers in Jhaukhel work in agriculture (i.e., their occupation increased physical activity). Nonetheless, peri-urban people tend to be less physically active in Nepal and in other Asian countries [4, 36, 37]. Such similarity of two villages can be useful for comparison and assessment of impact of the health education intervention if one of the villages is chosen as intervention area and another—as control.

### Mothers’ perceived barriers for improving their practice on diet and physical activity

Although knowledge and attitude toward healthy diet and physical activity are insufficient to affect practice [38], behavioral theories suggest that many other factors do [39]. For example, different barrier factors in families and environment can influence women as they try to develop a positive practicefor themselves and their children. Many of our respondents lived in extended families with their father- or mother-in-law as the head of household. Taste preference of the senior family members with the decision-making power was often an important and common barrier [18, 40].

Our respondents frequently reported lack of leisure time, absence of parks and playgrounds, being busy caring for children and old people, feeling lazy, and being embarrassed to exercise or jog in front of others as barriers for physical activity. In Nepal, people usually spend their leisure time talking, watching TV, or playing games or cards [4]. Activity like a “morning walk” is often seen among men but much less among women. Because a woman traditionally is seen as a person who takes care of the household and its family members, mothers are usually busy cooking in the morning and caring for their children. We also explored possible facilitators for healthy lifestyle.

We believe that health promotion intervention targeting mothers can tackle the existing gaps on diet and physical activity. Thus exploring the possible barriers and supporting factors for lifestyle changing and addressing them in the intervention will stimulate positive changes in mothers and hence their children’ lifestyle.

### Children’s behavior as perceived by mothers

Other studies have established that a mother’s level of education influences her children’sdietary behavior and physical activity [31, 41, 42]. Our current study supports those findings. Similar to studies in Turkey and Egypt, we observed that children’s diet scores increased with increasing their mothers’ education level [43, 44]. However, our results show that children’s physical activity scores decreased as their mother’s education level increased, possibly due to lower physical activity of better educated mothers and their position as role models [45]. Additionally, better educated mothers tended to have higher income, which means they can afford to purchase electronic devices that subsequently increase children’s screen time and thereby decrease physical activity.

### Implication of this study for research and practice

To our knowledge, this is the first study conducted in Nepal to explore whether KAP affects diet and physical activity in mothers with young children as well as comparing mothers’ KAPand children’s behavior. Our previous studies on cardiovascular health among the adult JD-HDSS population provided a good platform for the current study, which focused on a particular group in the community (i.e., mothers of young children). Thus, in addition to the qualitative study among mothers with young children previously done in JD-HDSS [18], the current study contributes to the needs assessment of KAP regarding diet and physical activity among mothers. Furthermore, with this study we established a baseline for future intervention programs in the community, hence enabling comparison of two semi-urban villages of JD-HDSS on mothers’ demographic variables, mothers’ KAP and children’s behavior regarding diet and physical activity is essential. Additionally, information on association of mothers’ KAP and children’s behavior with mothers’ demographic variables as well as perceived barriers for healthy lifestyle will help to develop and implement future interventions.

### Limitations of this study

Our study has some limitations as we reported children’s behavior as perceived by their mothers and did not interview the children directly. Moreover, data on physical activity of the mothers, which was collected using GPAQ [24], may be biased because it was self-reported and not objectively measured. Additionally, many of our respondents worked in agriculture, which has seasonal variations (i.e., in certain months, people are busy in the fields and at other times they are relatively free from field work). Thus, data collected during the growing season might have affected our results regarding respondents’ physical activity level.

## Conclusions

Our community-based study demonstrated that mothers of young children in JD-HDSS poorly understand the concepts of healthy/unhealthy diet, and identified a gap between knowledge and practice of mothers regarding diet and physical activity. Our findings suggest that JD-HDSS could benefit from a health education intervention designed to address the dietary and physical activity aspects of mothers’ practice. Besides improving knowledge, such an intervention should address attitude and practical changes (e.g., improving mothers’ self-efficacy). Moreover, deeper understanding of mothers’ self-perceived barriers for healthy lifestyle could offer important insights to the contents of the intervention. The results presented here support a future health education intervention targeting mothers with young children because they are prominent role models in their children’s lives [45, 35, 46]. In the long term, future health education interventions could therefore favorably contribute to decrease morbidity and mortality from CVD in JD-HDSS by shaping healthy practice in early childhood.

## Supporting information

S1 File Dataset. Raw data of the study.
(SAV)

S2 File Study tool, english version. (PDF)

S3 File Study tool, nepali version. (PDF)
