# Supplementary material for: Knowledge, attitude and practice on diet and physical activity among mothers with young children in the Jhaukhel-Duwakot Health Demographic Surveillance Site, Nepal
Source: PLoS One. 2018 Jul 9;13(7):e0200329. doi: 10.1371/journal.pone.0200329 (PMC6037384; doi:10.1371/journal.pone.0200329)
Supplement: S2 File — (PDF) [file pone.0200329.s002.pdf]

HARDIC

Health promotion for healthy diet and physical activity and its impact on cardiovascular health: A community-based trial among young mothers in a semi-urban community of Nepal

Questionnaire (baseline survey)

I. Basic information

SN	Question	Answer	Code
1	Serial Number of the respondent		
2	Village	1. Duwakot<br>2. Jhaukhel	
3	Ward no.		
4	Street/Tole		
5	House no		
6	Phone number		
7	Name of the head of household		
8	Husband's name		
9	How many persons (adults and children) live permanently (6 months or more) in your household?	|__|__| persons	
10	How many children aged less than 15 years (including responding child live permanently in your household?	|__|__| children	

II. Demographic information

A. Respondent's (mother's) information

Now I am going to ask you some information about yourself.

SN	Question	Answer	Code
11	Name of the respondent		
12	What is your year of birth?		
13	Relation to the head of household	1. Head<br>2. Wife<br>3. Mother-in-law<br>4. Mother<br>5. Daughter-in-law<br>6. Other (specify) ______________________ ☐	
14	What is your religion?	1. Hindu<br>2. Buddhism<br>3. Christian<br>4. Islam<br>5. Others (Specify) _____________ ☐	
15	What is your ethnicity?	1. Newar<br>2. Hill Brahmin<br>3. Chhetri<br>4. Tamang<br>5. Dalit<br>6. Thakuri<br>7. Magar<br>8. Rai<br>9. Others(Specify)______________ ☐	
16	What is the highest level of education you have completed?	1. Cannot read and write<br>2. No formal schooling but can read and write<br>3. Less than primary school (<1 class)<br>4. Primary school (class 3) completed<br>5. Secondary school (class 10) completed<br>6. High school (certificate /+2) completed<br>7. Graduation(bachelor) completed<br>8. Post graduate (masters) degree<br>9. PhD level ☐	
17	What is your main occupation?	1. Agriculture<br>2. Government employee<br>3. Labor<br>4. Student<br>5. Own business/sale<br>6. Housewife ☐	

		7. Private company<br>Others (specify)________________________	
18	Do you work now?	1. Yes<br>i. How long you are stay outside home every day(except weekend)?______________ hours<br>2. No	
19	What is your marital status?	1. Married<br>2. Separated<br>3. Divorced<br>4. Widow<br>5. Unmarried<br>☐	
20	What type of family do you have?	1. Nuclear<br>2. Joint<br>☐	
21	During the previous year, what was the household's average month income?	____________________ NRs	
22	What is your husband's main occupation?	1. Agriculture<br>2. Government employee<br>3. Labor<br>4. Student<br>5. Own business/sale<br>6. Private company<br>7. Retired<br>8. Others (specify)_________________<br>☐	

B. Children's information

> *Now I am going to ask you some information about your children.*

SN	Question	Answer	Code
23	How many children aged 1 to 7 do you have?<br><br>(*Write number in the square)*	☐	
24	Children information		

	(aged 1-7 yr)	1. 1.1.Name ______________________<br>1.2. Year and month of birth ______________________<br>1.3. Sex<br>1.4. Class completed____________________<br><br>2. 2.1. Name ___________________<br>2.2. Year and month of birth______________________<br>2.3. Sex<br>2.4. Class completed _________________<br><br>3. 3.1. Name ____________________<br>3.2. Year and month of birth______________________<br>3.3. Sex<br>3.4. Class completed _____________________	
25	Who does take care of your child most of the time?<br><br>*(Do not read all options to the respondent. Ask question and wait for an answer. Put number in the bracket accordingly)*	1. Mother and father<br>2. Mother<br>3. Father<br>4. Grandparents<br>5. Other relatives<br>6. Caretaker<br>7. Others(specify)______________________ ☐	

III. Knowledge of the mother

A. Diet

I am going to ask you now some questions regarding food and your dietary habits.

SN	Question	Answer	Code
26	What do you consider to be a healthy dietary habit for you?<br><br>**(yes/ no response for each)**<br><br>*(Read each option to the*	**Answer**<br>1. Large quantity of food ☐<br>**2.** Lot of fresh food ☐<br>*3.* Large variety of food ☐<br>*4.* Eating hygienic food ☐	

	respondent and ask her to answer yes/no. Put "Y" and "N" according answer in the box)	irrespective of the content<br>5. Eating main meals regularly and not snacking in between [ ]<br>6. Less sugar/sugary foods [ ]<br>7. Lot of vegetables [ ]<br>8. Eating smaller portions [ ]<br>9. Using less fat in cooking [ ]<br>10. Lot of fruit/ fresh juice [ ]	
27	I will give you some examples of food. What do you think is healthy food and what is not?<br><br>**(yes/ no response for each)**<br><br>*(Read each option to the respondent and ask her to answer yes/no. Put "Y" and "N" according answer in the box)*	**Answer**<br>1. Salad [ ]<br>2. All food which is available [ ]<br>3. Deep fried vegetables [ ]<br>4. Sausage, hamburgers, pizza [ ]<br>5. Sweetened soft drinks (Fanta, coca-cola, pepsi etc) [ ]<br>6. Fruits [ ]<br>7. Doughnuts, cookies, candies [ ]<br>8. Jam, Desserts [ ]<br>9. Chips, kurkure [ ]<br>10. Rice, vegetables, dal [ ]<br>11. Meat thukpa [ ]<br>12. Wai-wai soup [ ]<br>13. Roti, vegetables [ ]<br>14. *Chiura* [ ]<br>15. Traditional sweets (Sel, malpwa, jerry, etc) [ ]	
28	I will mention some disease now. Do you think developing of these conditions is associated with	**Answer** **Score**<br>1. Bad teeth [ ]<br>2. Injuries [ ]<br>3. Diabetes [ ]	

	unhealthy diet? Answer "Yes" or "No" for each condition.<br><br>Do you know health risk associated with unhealthy food?<br><br>**(Yes/No response for each)**<br><br>*(Read each option to the respondent and ask her to answer yes/no. Put "Y" and "N" according answer in the box)*	4. Leprosy<br>5. Heart diseases<br>6. High blood pressure<br>7. Rabies<br>8. Malaria<br>9. HIV/AIDS<br>10. Cancer	
29	Do you think that food we eat affect our heart?<br><br>*(Ask question and wait for answer. If it is difficult for respondent to respond read the given options)*	1. Yes<br>2. No<br>3. Have not thought about it<br>4. I don't care<br>5. I don't know<br>6. Others (specify)____________________	
30	What is junk food?<br><br>*(Ask respondent to give any 3 statements regarding junk food. It maybe food examples or some characteristic)*	1. ____________________<br>2. ____________________<br>3.<br>____________________	
31	Do you think your family consumes healthy food at home?	1. Very healthy<br>2. Quite healthy<br>3. Not very healthy<br>4. Very unhealthy<br>5. Don't know	

32. Healthy diet should contain:

I am going to ask your opinion regarding role of different food components for health. What amount (high or low) of each component should be present in healthy diet?

Items	A. High content	B. Low content	C. Amount is not important for health	D. Don't know	Code
32.1 Salt					
32.2 Sugar					
32.3 Grain foods					
32.4 Fruits					
32.5 Vegetables					
32.6 Fat					

B. *Physical activity*

Now I am going to ask you what you know about physical activity.

SN	Question	Answer	Code
33	What is the role of physical activity for health?<br>*Ask respondent to give any for statements according her understanding.*	1. ____________________<br>2. ____________________<br>3. ____________________<br>4. ____________________	
34	What are the effects of physical activity on our health?<br><br>**(Yes/No response for each)**<br><br>*(Read each option to the respondent and ask her to answer yes/no. Put "Y" and "N" according answer in the box)*	**Answer**<br>1. Improve physical health [ ]<br>2. Deteriorate our physical health [ ]<br>3. Improve mental health [ ]<br>4. Help fight with diseases [ ]<br>5. Cause injuries [ ]<br>6. Cause depression and frustration [ ]<br>7. Bad for the heart [ ]<br>8. Help us to look better [ ]<br>[ ]	

		9. Help avoid injuries<br>10. Nothing/none/no effect ☐<br>*(If answer is Yes, go to Q.35*	

35. What is the role of physical activity in the developing of the following diseases?

I will now give you examples of some diseases. Please tell me do you think physical activity is important in developing those diseases or not.

Put tick (√) in the appropriate box.

**Condition**	**A. Important**	**B. Not important**	**C. Don't know**	**Code**
35.1 Diabetes				
35.2 Cancer				
35.3 HIV/AIDS				
35.4 Gastritis				
35.5 Obesity				
35.6 Cataract				
35.7 High blood pressure				
35.8 Brain tumor				
35.9 Appendicitis				

IV. Attitude of mothers

A. Diet

36. I will read you some statements. Please, give your opinion regarding those statements. You are "strongly agree,"agree," disagree", "strongly disagree" or "neutral".

Give your opinion regarding statements given below:

Put tick (√) in the appropriate box. The answer can be only one for each statement.

**Statements**	**A. Strongly Agree**	**B Agree**	**C. Disagree**	**D. Strongly disagree**	**E. Neutral**	**Code**
36.1Healthy food is important						
36.2 Healthy foods are enjoyable						

36.3 I really care about what I eat						
36.4 Healthy food is not tasty						
36.5 Healthy food is for sick people						
36.6 If you do enough exercise you can eat whatever you like						
36.7 If you don't have any symptoms you can eat whatever you like						
36 .8 Tastiest foods are the ones that are bad for you						
36.9I should do a lot more about me and my children's health						
36.10 I wouldn't let my children eat junk food						
36.11 I always think of the calories in what I eat						
36.12 Soft drinks are good in the summer						
36.13 I buy soft drinks for my child because they are good for health						
36.14 When I become ill it's a matter of fate.						
36.15 I can stay healthy by taking good care of myself.						

SN	Question	Answer	Code
37	I will tell you different types of food one by one. Which food items out of them you consider to be tasty for you?<br><br>**(Yes/No response for each)**<br><br>*(Read each option to the respondent and ask her to answer yes/no. Put "Y" and "N" according answer in the*	**Answer**<br>1. Deep fried vegetables<br>2. Sausage, pizza<br>3. Sweetened soft drinks (Fanta, coca-cola, pepsi)<br>4. Fruits<br>5. Doughnuts, cookies, candies<br>6. Jam, Deserts<br>7. Chips, kurkure	

	box)	8. Rice, vegetables, dal<br>9. Meat thukpa<br>10. Wai-wai<br>11. Roti, vegetables<br>12. Salad	
38	Where do you get your nutrition information?<br><br>**(Multiple answer question)**<br><br>*(Ask question and wait for answer. If it is difficult for respondent to respond read the given options)*	1. Own mother, mother in law, other relatives<br>2. Friends, neighbors<br>3. TV, newspapers<br>4. Books<br>5. Internet<br>6. Others (specify)____________________<br>7. I don't have nutrition information<br>8. Don't know	
39	If you feel you have inadequate knowledge regarding which food is good or bad for health would you try to find such information yourself?<br><br>*(Ask question and wait for answer. If it is difficult for respondent to respond read the given options)*	1. Yes, I will ask someone who knows<br>2. I will try to read it somewhere<br>3. If the doctor hasn't told me anything it means it s not important<br>4. No, I don't know where to search and whom to ask<br>5. No, I can manage my food style without those knowledge<br>6. Never thought about it before<br>7. Others(specify)________________________<br>8. Don't know	
40	Do you want to improve your family's food pattern?<br><br>*(Circle the answer)*	1. Yes<br>i. If yes, why?_________________________<br>__________________________________<br>ii. What changes would you like to make?<br>__________________________________<br>__________________________________<br>2. No<br>i. If no, why? _______________________	

		___________________________________<br><br>3. Don't know	
41	What difficulties might you have with eating more healthy food?<br><br>**(Multiple answer question)**<br><br>*(Ask question and wait for answer. If it is difficult for respondent to respond read the given options)*	1. No difficulty trying to eat healthier<br>2. Cooking skills<br>3. Don't know enough about healthy eating<br>4. Busy lifestyle<br>5. Taste preferences of household members<br>6. I don't want to give up foods that I like<br>7. Price of healthy foods<br>8. Lack of will power<br>9. Others (specify)________________________<br>10. Don't know	
42	Which factors could support you to eat more healthy food?<br><br>**(Multiple answer question)**<br><br>*(Ask question and wait for answer. If it is difficult for respondent to respond read the given options)*	1. Own ill health<br>2. Advice from a doctor or nurse<br>3. Disease or death of a close relative<br>4. More money/ healthier food being less expensive<br>5. Better shops in the local area<br>6. Family members eating healthier food<br>7. Better information about food/ healthy eating<br>8. Nothing<br>9. Something else (specify)__________________________<br>10. Don't know	
43	Do you want to change your child food pattern?<br><br>*(Circle the answer)*	1. Yes<br><br>i. If yes, why?________________________<br>________________________________<br><br>iii. What changes would you like to make?<br>________________________________<br>________________________________	

		2. No<br>ii. If no, why? ______________________<br>__________________________________<br>3. Don't know	
44	Do you think that when children feel upset/bad mood, a favorite food will make them feel better?	1. Yes<br>2. No<br>3. Don't know<br>☐	
45	Do you think that if you love your child you should please him/her by buying favorite sweets?	1. Agree<br>2. Do not agree<br>3. Don't know<br>☐	
46	What do you think affects child's food choice?<br><br>**(Multiple answer question)**<br><br>*(Ask question and wait for answer. If it is difficult for respondent to respond read the given options)*	1. Family's food preferences<br>2. Mother's food preferences<br>3. Teaching in the school<br>4. Advertisement<br>5. Child's friends<br>6. Neighbors<br>7. Nothing is affecting<br>8. Others (specify)____________________<br>9. Don't know<br>☐	

47. Comment following statements:

I will read you some statements. Please, give your opinion regarding those statements. You are "agree," "moderately agree," moderately disagree", "disagree" or "neutral".

Put tick (√) in the appropriate box. The answer can be only one for each statement.

**Statements**	**A. Agree**	**B Moderately agree**	**B. Moderately disagree**	**D- Disagree**	**E- Neutral**	**Code**
47.1 . Media food advertising assists parents in their efforts to feed their child a healthy and balanced diet.						

47.2. Media food advertising leads children and their parents to spend their money on unnecessary and sometimes even harmful food products.						
47.3. Media food advertising is largely responsible for the weight problems and bad teeth of many children.						

B. *Physical activity*

I am going to ask your opinion regarding physical activity.

48. Give your opinion regarding statements given below:

I will read you some statements. Please, give your opinion regarding those statements. You are "strongly agree","agree," disagree", "strongly disagree" or "neutral ".

Put tick (√) in the appropriate box. The answer can be only one for each statement.

**Statements**	**A. Strongly Agree**	**B.- Agree**	**C Disagree**	**D Strongly disagree**	**E Neutral**	**Code**
48.1 Physical activity is important for children´s health						
48.2 Overweight children are more attractive						
48.3 Overweight children are healthier						
48.4 I am enjoying physical activity						
48.5 Physical activity is not important for adults						
48.6 Low physical activity is linked to overweight and						

obesity						
48.7 I eat less hence I don't need to be physically active						
48.8 I don't want to do more physical activity because it is increases my appetite and increase my weight						
48.9 After been diagnosed with heart disease one should restrict his/her physical activity						
48.10 Overweight people are more attractive						
48.11 The school has a responsibility for making sure my child gets enough exercise						
48.12. I restrict my child playing outside for safety reasons						
48.13. Parents have the main responsibility for making sure their child gets enough exercise						
48.14. Children's TV, electronic games and computer use should be restricted by their parents						

SN	Question	Answer	Code
49	If you need to reach some place which is more than 20-30 min walking distance what would you prefer?<br><br>*(Ask question and wait for answer. If it is difficult for respondent to respond read the given options)*	1. Cycling<br>2. Motorbike<br>3. Walking<br>4. Local bus<br>5. I will not go because it is too far<br>6. Others (Specify)____________________<br>7. Don't know	
50	How would you rate your physical activity status?	1. Very physically active<br>2. Fairly physically active<br>3. Not very physically active<br>4. Not at all physically active	

		5. Don't know	
51	Why did you choose to be physically active?	1. To maintain good health<br>2. My regular duty<br>3. To get or feel fit<br>4. Others (specify)________________________<br>5. Nor physically active<br>6. Don't know<br>☐	
52	Do you want to change your physical activity?	1. Yes<br>i. If yes, why?__________________________<br>___________________________________<br>ii. What changes would you like to make?<br>___________________________________<br>___________________________________<br>2. No<br>iii. If no, why? ________________________<br>_____________________________________<br>3. Don't know	
53	What are barriers for you to participate in physical activity?<br><br>**(Multiple answer questions)**	1. Lack of leisure time<br>2. Caring for children or older people<br>3. Lack of parks or other places for physical activity<br>4. Not feel the need to do more physical activity<br>5. Having other things to do<br>6. Own ill health<br>7. Feeling lazy<br>8. Feeling embarrassed in front of others<br>9. Others (specify)_________________________<br>10.Don't know<br>☐	
54	What are the factors facilitating you to be physically active?<br><br>**(Multiple answer questions)**	1. Own ill health<br>2. Advice from a doctor or nurse<br>3. Disease or death of a close relative<br>4. Support by friends, relatives<br>5. Better information regarding importance of physical activity<br>6. Availability of special places (parks, playgrounds etc.)<br>7. Nothing<br>☐	

		8. Something else (specify)________________<br>________________<br>9. Don't know	
55	How would you rate your child's physical activity status?	1. Very physically active<br>2. Fairly physically active<br>3. Not very physically active<br>4. Not at all physically active<br>5. Don't know	☐
56	Do you want to change your child's physical activity?	1. Yes<br>i. If yes, why?________________<br>________________<br>ii. What changes would you like to make?<br>________________<br>________________<br>2. No<br>i. If no, why? ________________<br>________________<br>3. Don't know	
57	What are barriers for your child to be physically active?<br><br>**(Multiple answer question)**	1. Too much homework<br>2. Lack of place to play outside the house<br>3. No friends to play with<br>4. Too dangerous to let child to play outside<br>5. My child has no barriers<br>6. Others(specify)________________<br>7. Don't know	☐
58	What are the factors facilitating your child to be physically active?<br><br>**(Multiple answer question)**	1. Physical activity is encouraged by parents<br>2. Availability of playgrounds outside<br>3. Parents and child spend time playing together<br>4. More free time<br>5. Friends<br>6. Load-shedding of electricity<br>7. Others (Specify)________________<br>8. Don't know	☐
59	Do you think you can influence	1. Yes	☐

	your child's physical activity?	2. No<br>3. Don't know	

III. Behavior of mothers and children

A. Family dietary pattern

SN	Question	Answer	Code
60	Who is usually cooking in your family?	1. Yourself<br>2. Husband<br>3. Father in law<br>4. Mother in law<br>5. Servant<br>6. Others (specify) ____________________	
61	Who is making decision regarding way of cooking and what to cook?	1. Yourself<br>2. Husband<br>3. Father in law<br>4. Mother in law<br>5. Servant<br>6. Children<br>7. Others (specify)____________________	
62	How often is your family eating out together?	1. Every day<br>2. Every alternate day<br>3. Once a week<br>4. Once a month<br>5. Never<br>6. Others (specify) ____________________<br>7. Don't know	
63	Do you eat differently during the weekend compared to the week?	1. Same food as on others days<br>2. Cooking something special<br>3. Going eating to relative's, friend's house<br>4. Going out (café, pasal, restaurant)<br>5. Others (specify)____________________<br>6. Don't know	

64	Do you give your child the same food which you eat usually at home?	1. Yes<br>2. No<br>i. If No, why?<br>______________________<br>3. Others (specify)______________<br>4. Don't know<br>☐	
65	Are sweetened soft drinks available at home during meals?	1. Yes, often or always<br>2. Sometimes<br>3. No or seldom<br>4. Don't know<br>☐	
66	Do you limit the amount of junk food at your home?	1. Don't know what junk food is<br>2. Try to keep healthy food at home but still have some unhealthy food<br>3. Don't care which food to give to the family<br>4. Having only healthy food at home<br>5. Others (specify)______________<br>6. Don't know<br>☐	

B.Child's dietary pattern

SN	Question	Answer	Code
67	What does your child eat during school time?	1. Taking tiffin from home every day<br>2. Sometimes eat at school sometimes take tiffin from home<br>3. Eat only school food<br>4. Buying food from nearby shops<br>5. Others (specify)______________<br>6. Doesn't attend school<br>7. Don't know<br>☐	
68	Where does your child eat healthiest food?	1. At home<br>2. At school<br>3. Out<br>☐	

		4. Other (specify) ____________________<br>5. Don't know	
69	How often does your child get pocket money?	1. Every day<br>2. Two-three times a week<br>3. Once a week<br>4. Never<br>5. Others (specify)____________________<br>6. Don't know	
70	How does your child usually spend them?<br><br>(**Multiple answer question)**	1. Buying some food<br>2. Buying chocolates<br>3. Buying drinks (coke, fanta etc)<br>4. Buying toys<br>5. Playing games in the internet cafe<br>6. Saving<br>7. Others (specify)____________________<br>8. I don't care<br>9. I don't give pocket money to my child<br>10. Don't know	

71. What types of foods does your child usually eat as a snack?

**Items**	**A. Often**	**B. Sometimes**	**C. Seldom**	**Code**
71.1. Chaomin				
71.2. Wai-wai				
71.3. Homemade vegetables and roti				
71.4. Chura, meat or vegetables				
71.5. Biscuits, bread				
71.6. Sweets				
71.7. Chaatpate, samosa, pani puri				
71.8. Momo				
71.9. Egg				
71.10. Milk				

71.11. Dahi				
71.12. Fresh salad (carrot, cucumber, mula)				
71.13. Others (specify)				

SN	Question	Answer	Code
72	Name 3 most favorite food items of your child.	1. ________________<br>2. ____________________<br>3. ____________________<br>4. Don't know	
73	What are your child's favorite drinks?<br>**(Multiple answer question)**	1. Water<br>2. Milk<br>3. Tea<br>4. Real juice, Slice<br>5. Soft drinks (coca cola, fanta, dew, sprite)<br>6. Fresh juice<br>7. Horlicks, Complan<br>8. Tang<br>9. Others (specify)_______________<br>10. Don't know	
74	How often does your child consume soft drinks?	1. Every day<br>2. Twice a day<br>3. Twice a week<br>4. Once a week<br>5. Never<br>6. Others (specify)__________________<br>7. Don't know	
75	Does your child eat while watching TV?	1. Yes<br>i. If yes, how often? ______days/week<br>2. No *(go to Q. 77)*<br>3. Don't know	
76	If yes, which food/drinks does he/she prefer to eat while watching TV, playing computer	1. Fruits<br>2. Cookies<br>3. Chips, kurkure, wai-wai	

	games?<br><br>**(Multiple answer qurstion)**	4. Candy, chocolates<br>5. Nuts, dry fruits<br>6. Soft drinks<br>7. Others (specify)____________________<br>8. Don't know	
77	Does your child ask you to buy food items he/she saw on TV?	1. Never<br>2. Sometimes<br>3. Often<br>4. My child hardly watches TV<br>5. Don't know<br>☐	
78	Suppose you are in a market/shop, and your child asks for a specific food item that you did not think of buying initially, do you buy it?	1. Usually not<br>2. Sometimes<br>3. Often<br>4. Only if I have the money<br>5. Only if it is something healthy<br>6. Don't know<br>☐	
79	Where does your child usually have breakfast?	1. Home<br>2. School<br>3. On the way to school (shops, café, street vendors)<br>4. Others (specify)_____________________<br>5. Don't know<br>☐	
80	What does your child usually eat at breakfast?	1. ______________________<br>2. ______________________<br>3. ______________________	
81	What does your child usually eat at dinner?	1. ______________________<br>2. ______________________	

C. Physical activity

82. Mother's vigorous activities (for at least 10 minutes at time)

**82.1 Work** (heavy lifting , digging or construction)	**Code**	**82.2 Leisure time** (running or strenuous sports, weight lifting)	**Code**
Yes No<br>[ ] NA<br>**Number of days/week** *go to **82.2***<br>Hours [ ]<br>min [ ]<br>**How much time**	**82.1a**<br>**82.1b**<br>**82.1c**<br>**82.1d**	Yes No<br>[ ] NA<br>**Number of days/week** *go to **83.1***<br>Hours [ ]<br>min [ ]<br>**How much time**	**82.2.a**<br>**82.2b**<br>**82.2c**<br>**82.2d**

83. Mother's moderate intensity activities (for at least 10 minutes at time)

**83.1 Work** (brisk walking or carrying light loads, housework)	**Code**	**83.2 Leisure time** (brisk walking, cycling)	**Code**
Yes No<br>[ ] NA<br>**Number of days/week** *go to **82.2***<br>Hours [ ]<br>min [ ]<br>**How much time**	**83.1a**<br>**83.1b**<br>**83.1c**<br>**83.1d**	Yes No<br>[ ] NA<br>**Number of days/week** *go to **83.1***<br>Hours [ ]<br>min [ ]<br>**How much time**	**83.2.a**<br>**83.2b**<br>**83.2c**<br>**83.2d**

**SN**	**Question**	**Answer**	**Code**

84	How much time you usually spend sitting or reclining on typical day?	__________ hours/day	
85	Does your child attend school?	1. Yes<br>2. No *(go to Q. 88)*	
86	How does your child usually get to school?	1. Walking<br>2. School bus<br>3. Car, motorbike<br>4. Cycling<br>5. Others (specify)____________________<br>6. Don't know	
87	How long does your child walk (including walking to and from the bus stop) for going to and coming back from the school?	________________ min/day	
88	How does your child like to spend spare time (after homework is done or on the vacation)?<br><br>**(Multiple answer question)**	1. Read books<br>2. Playing alone at home<br>3. Playing with friends at home<br>4. Playing outside<br>5. Watching TV, computer or mobile games<br>6. Others (specify)____________________<br>7. Don't know	
89	How much time does your child usually spend per day doing homework?	__________ min/day	
90	How much time does your child usually spend per day playing in the yard or street around your house (or the house of a friend, neighbour or relative) on the typical weekdays?	__________ min/day	

91	How much time does your child usually spend per day playing in the yard or street around your house (or the house of a friend, neighbour or relative) on the weekends or holidays?	_________ min/day	
92	How much time does your child spend using computers, mobile games, TV per day on weekdays?	_________ min/day	
93	How much time does your child spend using computers, mobile games, TV on weekends or holidays?	_________ min/day	

THANK YOU
