# Supplementary material for: Knowledge, attitude and practice on diet and physical activity among mothers with young children in the Jhaukhel-Duwakot Health Demographic Surveillance Site, Nepal
Source: PLoS One. 2018 Jul 9;13(7):e0200329. doi: 10.1371/journal.pone.0200329 (PMC6037384; doi:10.1371/journal.pone.0200329)
Supplement: S3 File — (PDF) [file pone.0200329.s003.pdf]

Baseline assessment of Knowledge, Attiitude and Behaviour about Diet and Physical Activity among mothers in a semi-urban community of Nepal

स्वस्थ खानपान तथा शारीरिक क्रियाकलाप बारेआमाहरुमा सर्वेक्षण

QUESTIONNAIRE (BASELINE SURVEY) प्रारम्भिक सर्वेक्षण प्रश्नावली

अन्तरवार्ता लिनेको कोड नं. :...

अन्तरवार्ता शुरु गरेको समय: ……….……… अन्तरवार्ता अन्त्य भएको समय : ……….…………..

अन्तरवार्ताकार	फिल्ड सुपरभाइजर	तथ्याङ्क भर्ने
मिति:………………..	मिति:………………..	मिति:………………..

I. **सूचना फारम सम्बन्धि तथा आधारभूत विवरण जानकारी**

क्र.सं.	विवरण						कोड
**1.**	उत्तरदाता (सहभागी) क्रम संख्या	**VDC**	**Ward**	**House no.**		**E. Code**	G1
**2.**	गाउँ वा टोलको नाम						G2
**3.**	चिठ्ठा प्रणालीबाट छानिएको उत्तरदाता	**हो**		**होइन**			G3
**4.**	घरको नक्सा						G4
**6.**	फोन नं. :						G5
**7.**	घरमुलीको नाम :						G6
**8.**	श्रीमान् (पति)को नाम :						G7
**9.**	यस घरधुरीमा जम्मा कति जना मानिसहरु (वयश्क र बालबालिका समेत) बस्दछन् ? (६ महिना वा सोभन्दा बढी बसेको हुनुपर्छ)	☐☐ जना					G8
**10.**	यस घरधुरीमा कति जना १५ वर्ष मुनिका बालबालिकाहरु (सहभागी उत्तरदाता बालबालिका समेत) बस्दछन् ?	☐☐ जना बालबालिकाहरु					G9

आधिकारिक मञ्जुरीनामा (प्रथम चरण)

नमस्कार,

मेरो नाम .... हो र म स्वास्थ्य तथा जनसाँखिक सर्वेक्षण क्षेत्र परियोजना (JD-HDSS Project) काठमाडौ मेडिकल कलेजको तर्फबाट यहाँ आएको हुँ । यो परियोजना भक्तपूर जिल्लाको दुई गा.वि.स.हरु दुवाकोट र झौखेलमा सञ्चालित छ । यसै परियोजना अन्तर्गत “ **स्वस्थ खानपान तथा शारीरिक क्रियाकलाप बारे आमाहरुमा सर्वेक्षण** ” अध्ययनको सिलसिलामा हामीले यस जिल्लाको दुई गाविसहरुमा आधारभूत सर्भेक्षण गर्न लागेका छौँ । यस सर्वेक्षणको मुख्य उद्देश्य भनेको खानपान र शारीरिक क्रियाकलाप वा अभ्यास प्रति तपाईको ज्ञान, अवधारणा तथा व्यवहार मापन गर्नुका साथै तपाईसँग भएको ज्ञान वा सिप कसरी आफ्नो बालबालिकाहरुका लागि प्रयोग गरिरहनु भएको छ त्यो पनि हेर्नु हो । यस सर्वेक्षण अन्तर्गत हामी तपाईको र तपाईको बच्चाहरुको उचाइ र तौल समेत लिनेछौं ।

यस सर्भेक्षणमा तपाईको सहभागिताले स्वास्थ्य सम्बन्धी जनचेतना जगाउन तथा रोगहरुको रोकथाम गर्न मद्धत मिल्नेछ । हामी तपाईलाई यस सर्भेक्षणमा भएका प्रश्नहरु सोध्न चाहन्छौ जुन करीब ४० मिनेट समय लाग्नेछ ।

यस सर्भेक्षणको सिलसिलामा म तपाईको र तपाईको बच्चाहरुको बारेमा प्रश्नहरु सोध्नेछु । तपाईले दिनु भएको सूचनाहरु वा जानकारीले कसैलाई हानी गर्ने छैन साथै तपाईको नाम र यी जानकारीहरुलाई अति गोप्य राखिनेछ , कसैलाई भनिने छैन । यो सर्भेक्षणमा भाग लिने वा नलिने तपाईको पूर्ण मन्जुरीको आधारमा हुनेछ । यदी तपाईलाई सोधेको प्रश्नको उत्तर दिन नचाहेमा मलाई भन्नु होला, र म तपाईलाई अर्को प्रश्न सोध्नेछु वा तपाईले चाहको बेलामा यो अन्तरवार्ता अन्त्य गर्न सक्नुहुनेछ । यस सर्भेक्षणमा तपाईको विचार निकै महत्वपूर्ण छ र मलाई आशा छ तपाईले अवश्य सहयोग गर्नुहुनेछ । तपाईलाई यस सर्वेक्षण सम्बन्धी केही जिज्ञाशा भए सोध्न सक्नुहुनेछ ।

यदि तपाईको केही जिज्ञाशा छैन भने, के तपाई यस अन्तरवार्तामा सहभागी हुन मञ्जुर हुनुहुन्छ ?

हुन्छ		हुँदैन	

के तपाई यस मञ्जुरीनामा सही गर्नुहुन्छ ?

हुन्छ , सही गर्छु		सही गर्दिन	

(यदि सहभागीले सही नगरे पनि फेरि सोध्नुहोस्) के तपाई यस अन्तरवार्तामा सहभागी हुन मञ्जुर हुनुहुन्छ ? के म अब तपाईलाई प्रश्नहरु सोध्न सक्छु ? *(मौखिक मञ्जुरी लिनुहोस्)*

हुन्छ		साक्षीको सही	
हुँदैन			

(यदि अन्तरवार्तामा सहभागी नहुँने भएमा पनि वहाँको समयको लागि सहभागीलाई धन्यवाद दिनुहोस्)

तपाईलाई धन्यवाद छ ।

सर्भेक्षण फारमको नतिजा

**नतिजा**	
**1.** पूर्णरुपमा फारम पूरा भएको<br>**2.** केही उत्तर वाहेक फारम पूरा भएको<br>**3.** लामो समयदेखि घरमा नभएको<br>**4.** फारम भर्न अस्विकार गरेको<br>**5.** अन्य भए उल्लेख गर्नुहोस...........................	☐

II. जनसांखिक विवरण

A. सहभागी उत्तरदाता (आमा) सम्बन्धी विवरण

अब म तपाईलाई तपाईकै बारेमा केही प्रश्नहरु सोध्नेछु ।			
**क्र.सं.**	**प्रश्नहरु**	**उत्तर**	**कोड**
**11.**	तपाईको पुरा नाम के हो ?<br>सहभागी (उत्तरदाता)को नाम :		D1
**12.**	तपाईंको जन्म कति सालमा भएको हो? (यदि उत्तरदाताले वर्षमा उत्तर दिन्छ भने वर्षलाई २०७१ मा घटाएर जन्मेको साल लेख्नुहोस)	वि.स……………………. साल	D2
**13.**	घरमुलीसँग सहभागी (उत्तरदाता) को नाता	1. आफै घरमुली<br>2. घरमुलीको श्रीमती वा पत्नी<br>3. घरमुलीको बुहारी<br>4. घरमुलीको सासू<br>5. घरमुलीको आमा<br>6. अन्य (उल्लेख गर्ने) ____________ ☐	D3
**14.**	तपाईं कुन धर्म मान्नुहुन्छ ?	1. हिन्दू<br>2. बौद्ध<br>3. इशाई<br>4. इस्लाम<br>5. अन्य (उल्लेख गर्ने)______________ ☐	D4
**15.**	तपाईंको जाती के हो ? वा तपाईं कुन जातीको हो ?	1. नेवार<br>2. पहाडी ब्राम्हण<br>3. छेत्री<br>4. तामाङ्ग<br>5. दलित<br>6. ठकुरी<br>7. मगर<br>8. राई<br>9. अन्य (उल्लेख गर्ने)_______________ ☐	D5
**16.**	तपाईले कति (कक्षा)सम्म पढ्नुभएको छ?	1. लेख्न पढ्न नसक्ने<br>2. लेखपढ गर्न सक्ने तर विद्यालय नगएको<br>3. विद्यालय गएको तर प्राथमिक तह (कक्षा ५) पुरा नगरेको<br>4. प्राथमिक तह (कक्षा ५) पुरा गरेको<br>5. माध्यमिक तह (कक्षा १०) पुरा गरेको<br>6. प्रमाणपत्र तह (कक्षा १२) पुरा गरेको<br>7. स्नातक तह (बि.ए. सरह) पुरा गरेको<br>8. स्नातकोत्तर तह (एम.ए. सरह) पुरा गरेको<br>9. विद्यावारिधी तह (पीएच.डी) पुरा गरेको<br>10. अन्य (उल्लेख गर्ने)_______________ ☐	D6
**17.**	तपाईको पेशा के हो ? वा तपाई आय आर्जन हुने के काम गर्नुहुन्छ?	1. कृषि<br>2. सरकारी जागिर<br>3. श्रम गरेर खाने वा ज्यालादारी<br>4. विद्यार्थी<br>5. आफ्नै व्यवसाय<br>6. गृहिणी<br>7. निजी संस्थामा जागिरे<br>8. अन्य (उल्लेख गर्ने)_______________ ☐	D7

**18.**	के तपाई अहिले कतै काम गर्नुहुन्छ ?<br><br>*(**गर्छु** वा **गर्दिन** मा घेरा लगाउनुहोस्)*	1. गर्छु<br>i. यदि काम गर्नुहुन्छ भने दैनिक कति समय घरबाहिर रहनुहुन्छ (छुट्‌टीका दिन बाहेक?<br>………………… घण्टा ।<br>2. गर्दिन	D8<br><br><br>D8i
**19.**	तपाईको वैवाहिक अवस्था के हो?	1. विवाहित<br>2. श्रीमानसँग छुट्टिएर बसेको<br>3. पारपाचुके गरेको<br>4. विधवा<br>5. अविवाहित	D9
**20.**	तपाईको कस्तो प्रकारको परिवार हो?	1. एकल (सानो) परिवार<br>2. बृहत (ठुलो) परिवार	D10
**21.**	गएको वर्षको आधारमा भन्नु पर्दा तपाईंको घरको मासिक आम्दानी कति थियो होला ?	____________________ नेपाली रुपैंया प्रति महिना	D11
**22.**	तपाईंको श्रीमान् वा पतिको मुख्य पेशा (आम्दानीको श्रोत) के हो ?	1. कृषि<br>2. सरकारी जागिर<br>3. श्रम गरेर खाने वा ज्यालादारी<br>4. विद्यार्थी<br>5. आफ्नै व्यवसाय<br>6. निजी संस्थामा जागिरे<br>7. सेवा निवृत्त (उमेर पुगी जागिर सकिएको)<br>8. बैदेशिक रोजगार<br>9. अन्य (उल्लेख गर्ने)____________________________	D12

***B.* बालबालिकासम्बन्धी विवरण वा जानकारी**

**अब म तपाईलाई तपाईका बालबालिकाहरु (बच्चाहरु)को बारेमा केही प्रश्नहरु सोध्नेछु ।**			
यदि सहभागी (उत्तरदाता)को एकभन्दा बढी १ देखि ७ वर्षका बालबालिका छन् भने प्रत्येकलाई ठूलो देखी सानो हुदैं क्रमै सगं १, २, ३ मा राख्ने। यदि एउटा मात्र बच्चा भए बच्चा १ मात्र लेख्ने ।			
**क्र.सं.**	**प्रश्नहरु**	**उत्तर**	**कोड**
**23.**	तपाईंका १ देखि ७ वर्ष उमेरका कति जना बालबच्चाहरु छन् ?(संख्या अर्को कोठाको बाकसभित्र लेख्ने)	जना	Dc1
**24.**	बालबालिकाहरुको विवरण<br><br>*(उमेर १ देखि ७ वर्ष)*	**बच्चा १**<br>1. नाम : ____________________________<br>2. जन्म साल र महिना _______________<br>3. लिङ्ग : __________<br>4. उतिर्ण गरेको कक्षा : _____________<br>**बच्चा २**<br>1. नाम : ____________________________<br>2. जन्म साल र महिना_______________<br>3. लिङ्ग : __________<br>4. उतिर्ण गरेको कक्षा : _____________<br>**बच्चा ३**<br>1. नाम : ____________________________<br>2. जन्म साल र महिना_______________<br>3. लिङ्ग : __________<br>4. उतिर्ण गरेको कक्षा : _____________	Dc2.1.1<br>Dc2.1.2<br>Dc2.1.3<br>Dc2.1.4<br><br>Dc2.2.1<br>Dc2.2.2<br>Dc2.2.3<br>Dc2.2.4<br><br>Dc2.3.1<br>Dc2.3.2<br>Dc2.3.3<br>Dc2.3.4

**25.**	तपाईंको बालबच्चाको हेरचाह धेरैजसो कसले गर्छ ? <br>*(दायाँतिर भएका उत्तरहरु तुरुन्तै नभन्नुस्। प्रश्न सोधेपछि केही समय पर्खनुस् । यदि सहभागीलाई उत्तर भन्न गाह्रो भएमा मात्र उक्त उत्तरहरु पढ्नुहोला ।)*	1. आफैले 2. श्रीमान (पति)ले 3. आमाले/ सासुले 4. बुवाले/ ससुराले 5. हजुरबा हजुरआमाले 6. अन्य नातेदारले 7. बच्चा हेर्ने व्यक्तिले 8. अन्य (उल्लेख गर्ने) __________ ☐	Dc3

III. आमाको ज्ञान (चेतना) सम्बन्धी विवरण

***A.* खानपान**

अब म तपाईलाई खानपान सम्बन्धी तपाईको ज्ञान वा चेतना केकस्तो छ भनी सोध्नेछु ।

क्र.सं.	प्रश्नहरु	उत्तर	कोड
**26.**	तपाईको विचारमा स्वस्थ खानपान (को बानी) भन्नाले के बुझ्नुहुन्छ ? वा कस्तो बानीलाई तपाई स्वस्थ **खानपानको बानी** भन्नुहुन्छ ? <br>*(दायाँतिर भएका प्रत्येक उत्तर पढेर सुनाउनुहोस् र प्रत्येकमा **हो** वा **होइन** भनी सोध्नुहोस् । उत्तरदाताबाट आएको उत्तरलाई सँगैको बाकसभित्र **हो** भने **“1”** तथा **होइन** भने **“0”** र **थाहा छैन** भने **“99”**लेख्नुहोस् ।)*	**प्रश्न** / **उत्तर** 1. धेरै खाना 2. धेरै ताजा खाना 3. धेरै प्रकारका खाना 4. जति मात्रामा भए पनि सफा खाना खाने 5. साँझ विहान राम्ररी खानाखाने बीचबीचमा खाजा नखाने 6. चिनी कम भएको खाना 7. धेरै तरकारी (सब्जी) खाने 8. थोरै मात्रामा तर बारम्बार (घरिघरि) खाइरहने 9. पकाउँदा चिल्लो जस्तै तेल कम हाल्ने 10. प्रसस्त फलफुल तथा फलफुलको ताजा रस खाने	Kd1.1 Kd1.2 Kd1.3 Kd1.4 Kd1.5 Kd1.6 Kd1.7 Kd1.8 Kd1.9 Kd1.10
**27.**	तपाईलाई अब म केही **खानेकुराहरुको नाम** पढेर सुनाउनेछु । अब, तपाईले भन्नुहोस कि यी खानेकुरा स्वस्थकर **छ** कि स्वस्थकर **छैन**? <br>*(दायाँतिर भएका प्रत्येक खानेकुरा पढेर सुनाउनुहोस् र प्रत्येकमा स्वस्थकर **छ** वा **छैन** भनी सोध्नुहोस् । उत्तरदाताबाट आएको उत्तरलाई सँगैको बाकसभित्र स्वस्थकर **छ** भने **“1”**तथा **छैन** भने **“0”**र **थाहा छैन** भने **“99”**लेख्नुहोस् ।)*	**प्रश्न** / **उत्तर** 1. सलाद (गाजर, मुला, काँक्रा आदि) 2. तारेको तरकारी 3. ससेज, पीज्जा, बर्गर 4. गुलियो पेय पदार्थ जस्तै कोकाकोला 5. फलफुल 6. डोनट, बिस्कुटआदि 7. चकलेट 8. मिठाई (लड्डु, पेडा, बर्फी) 9. पारम्पारिक नेपालीरोटी(सेलरोटी, मालपुवा, रोठ) 10. केक 11. आलुचिप्स तथा कुरकुरे 12. दाल, भात, तरकारी 13. मासु 14. झोल भएको चाउचाउ 15. सुख्खा चाउचाउ 16. रोटी तरकारी 17. च्युरा 18. उपलब्ध हुने वा पाउने जति सबैप्रकारका खानेकुरा	Kd2.1 Kd2.2 Kd2.3 Kd2.4 Kd2.5 Kd2.6 Kd2.7 Kd2.8 Kd2.9 Kd2.10 Kd2.11 Kd2.12 Kd2.13 Kd2.14 Kd2.15 Kd2.16 Kd2.17 Kd2.18

**28.**	अब म तपाईलाई केही रोग वा विमारीहरुको बारेमा भन्नेछु र तपाईले मलाई भन्नुस् कि, के यी रोग वा विमारी अस्वस्थकर खानपान (खानेकुरा)सँग सम्बन्धित छ कि छैन?<br><br>*(दायाँतिर भएका प्रत्येक रोग वा विमारी पढेर सुनाउनुहोस् र प्रत्येकमा सम्बन्धि छ **छ** वा **छैन** भनी सोध्नुहोस् । उत्तरदाताबाट आएको उत्तरलाई सँगैको बाकसभित्र सम्बन्धित **छ** भने **"1"**तथा **छैन** भने **"0"** र **थाहा छैन** भने **"99"**लेख्नुहोस् ।)*	**प्रश्न** / **उत्तर**<br>1. दाँतको खराबी<br>2. चोटपटक लाग्ने<br>3. मधुमेह वा चिनीरोग हुने<br>4. कुष्टरोग लाग्ने<br>5. मुटुको रोग लाग्ने<br>6. उच्च रक्तचाप हुने<br>7. झाडापखाला<br>8. रेबिज लाग्ने (बहुला कुकुरले टोक्दा हुने रोग)<br>9. औलो (मलेरिया) लाग्ने<br>10. एचआइभी एड्स लाग्ने<br>11. अर्बुद रोग (क्यान्सर)	Kd3.1<br>Kd3.2<br>Kd3.3<br>Kd3.4<br>Kd3.5<br>Kd3.6<br>Kd3.7<br>Kd3.8<br>Kd3.9<br>Kd3.10<br>Kd3.11
**29.**	तपाईको विचारमा, के हामीले खाएको खानेकुराले मुटुलाई असर गर्छ कि गर्दैन ?<br>*(दायाँतिर भएका उत्तरहरु तुरुन्तै नभन्नुस् । प्रश्न सोधेपछि केही समय पर्खनुस् । यदि सहभागीलाई उत्तर भन्न गाह्रो भएमा मात्र उक्त उत्तरहरु पढ्नुहोला ।)*	1. गर्छ<br>2. गर्दैन<br>3. यसको बारेमा सोचेकै छैन<br>4. गर्छ तर म वास्ता नै गर्दिन<br>5. मलाई थाहा छैन<br>6. अन्य (उल्लेख गर्ने) __________	Kd4
**30.**	तपाईको बिचारमा **जंक फुड** भनेको के हो अनि के कस्ता खानेकुरालाई **जंक फुड** भन्नुहुन्छ ?<br>*(यो कुनै खानेकुरा वा खानेकुराको प्रकार हुनसक्छ । उत्तरदाताबाट आएको सबै उत्तरहरुलाई बेग्लाबेग्लै हरफमा वा लाइनमा लेख्नुहोला)*	1. ______________<br>2. ______________<br>3. ______________	Kd5.1<br>Kd5.2<br>Kd5.3
**31.**	तपाईको विचारमा, तपाईको परिवारले घरमा प्रायजसो खाने गरेको खाना वा खानेकुरा कत्तिको स्वस्थकर छ ?	1. एकदम स्वस्थकर छ<br>2. अलिअलि स्वस्थकर छ<br>3. त्यति स्वस्थकर छैन<br>4. एकदमै अस्वस्थकर छ<br>5. थाहा छैन वा भन्न सक्दिन	Kd6

32. स्वस्थकर खानपानमा तलका तत्वहरुको मात्रा कत्तिको हुनुपर्छ ?

म तपाईलाई स्वास्थ्यका लागि खानेकुराहरुमा पाइने विभिन्न प्रकारका तत्वहरुको भूमिका के हुन्छ भनी सोध्नेछु । स्वस्थ खानपानमा तल दिइएका तत्वहरुको मात्रा के कति थोरै वा धेरै हुनुपर्छ होला ?

**Items**<br>**तत्वहरु**	**A**<br>**धेरै मात्रामा चाहिन्छ**	**B**<br>**थोरै मात्रामा चाहिन्छ**	**C**<br>**ठिकै मात्रामा चाहिन्छ**	**D**<br>**थोरै-धेरै जति खाए पनि स्वास्थ्यको लागि खासै फरक पर्दैन**	**E**<br>**थाहा छैन**	**कोड**
**32.1** नून						Kd7.1
**32.2** चिनी						Kd7.2
**32.3** अन्न-गेडागुडी						Kd7.3
**32.4** फलफुल						Kd7.4
**32.5** सागसब्जी						Kd7.5
**32.6** मासुको बोसो, तेल, घ्यू , बटर, नौनी						Kd7.6

B. शारीरिक क्रियाकलाप वा अभ्यास

अब म तपाईलाई शारीरिक क्रियाकलाप वा अभ्यास भन्नाले तपाई के बुझ्नुहुन्छ भनी सोध्नेछु ।

क्र.सं.	प्रश्नहरु	उत्तर	कोड
**33.**	हाम्रो स्वास्थ्यमा शारीरिक क्रियाकलापको के भूमिका हुन्छ?<br><br>*(उत्तरदाता-सहभागीलाई तपाईले जे बुझ्नु भएको छ त्यही भन्दा हुन्छ भन्नुहोस्)*	1. ______________________<br>2. ______________________<br>3. ______________________<br>4. ______________________	Kp1.1<br>Kp1.2<br>Kp1.3<br>Kp1.4
**34.**	अब म तपाईलाई विभिन्न स्वास्थ्य स्थिति वा स्वास्थ्य अवस्थाहरुको बारेमा भन्नेछु । यीमध्ये कुन चाहीँ पर्याप्त मात्रामा शारीरिक अभ्यास (क्रियाकलाप)को कारणले गर्दा **हुन्छ** वा **हुँदैन** भन्नुहोस् ।<br>*(दायाँतिर भएका प्रत्येक स्वास्थ्य अवस्था पढेर सुनाउनुहोस् र प्रत्येकमा **हुन्छ** वा **हुँदैन** भनी सोध्नुहोस् । उत्तरदाताबाट आएको उत्तरलाई सँगैको बाकस भित्र **हुन्छ** भने **"1"** तथा **हुँदैन** भने **"0"**र **थाहा छैन** भने **"99"**लेख्नुहोस् ।)*	(see table below)	Kp2.1<br>Kp2.2<br>Kp2.3<br>Kp2.4<br>Kp2.5<br>Kp2.6<br>Kp2.7<br>Kp2.8<br>Kp2.9

**प्रश्न**	**उत्तर**
1. शारीरिक स्वास्थ्य राम्रो हुन्छ	
2. शारीरिक स्वास्थ्य बिग्रन्छ	
3. मानसिक स्वास्थ्य राम्रो हुन्छ	
4. रोगसँग लड्न मद्दत गर्छ	
5. घाउ वा चोटपटक लाग्छ	
6. दिक्क लाग्ने, उदासिन हुने वा चिन्तित हुने	
7. मुटुलाई हानी गर्छ	
8. आकर्षक देखिन मद्दत गर्छ	
9. चोटपटक लाग्नबाट जोगाउँछ	

35. म तपाईलाई रोगहरुका केही उदाहरणहरु बताउने छु । कुनैपनि मानिसले कत्तिको शारीरिक (क्रियाकलाप) अभ्यास गर्छ, त्यसले यी रोगहरुको विकास हुन मद्दत गर्छ कि गर्दैन?

(उत्तर मिल्ने कोठामा चिन्ह (√) लाउनुहोला)

**Condition**<br>**अवस्था**	**A**<br>**गर्छ**	**B**<br>**गर्दैन**	**C**<br>**थाहा छैन**	**कोड**
**35.1** मधुमेह वा चिनीरोग				Kp3.1
**35.2** अर्बुद रोग (क्यान्सर)				Kp3.2
**35.3** एचआइभी एड्स				Kp3.3
**35.4** ग्यास्ट्राईटिस				Kp3.4
**35.5** मोटोपना हुने				Kp3.5
**35.6** झाडा-पखाला				Kp3.6
**35.7** आँखाको नानीमा रोग (फुलो)				Kp3.7
**35.8** उच्च रक्तचाप				Kp3.8
**35.9** मष्तिस्कमा मासु पलाउने				Kp3.9
**35.10** एपेन्डिसाईटिस (पुच्छे आन्द्रा सुन्निने)				Kp3.10

IV. आमाको (अव)धारणा

***A.* खानपान**

अब म खानपान सम्बन्धी तपाईको सोचाइ के छ बुझ्न चाहन्छु ।

36. म केही भनाइहरु पढेर सुनाउनेछु । यी भनाइहरु प्रति तपाईको धारणा वा बिचार भन्नुहोस् । तपाई एकदमै सहमत वा अलिअलि सहमत वा असहमत वा एकदमै असहमत वा थाहा छैन भन्न सक्नुहुनेछ ।

(उत्तर मिल्ने कोठामा चिन्ह (√) लाउनुहोला तर प्रत्येक भनाइमा एउटा मात्र उत्तर हुनेछ ।)

**Statements** भनाइहरु		**A** एकदमै सहमत	**B** अलिअलि सहमत	**C** अलिअलि असहमत	**D** एकदमै असहमत	**E** थाहा छैन	कोड
**36.1**	स्वस्थकर खानेकुरा महत्वपूर्ण हुन्छ						Ad1.1
**36.2**	स्वस्थकर खानेकुरा आनन्दायी हुन्छ						Ad1.2
**36.3**	आफुले खाने खानेकुराको बारेमा सचेत हुनुपर्छ						Ad1.3
**36.4**	स्वस्थकर खानेकुरा स्वादिलो हुँदैन						Ad1.4
**36.5**	स्वस्थकर खानेकुरा बिरामीका लागि हो						Ad1.5
**36.6**	यदि शारीरिक अभ्यास गरिन्छ भने आफुलाई मन लागेको जे खाए पनि हुन्छ						Ad1.6
**36.7**	यदि कुनै रोगको लक्षण छैन भने आफुलाई मन लागेको जे खाए पनि हुन्छ						Ad1.7
**36.8**	स्वादिला खानेकुराहरुले स्वास्थ्यलाई खराब गर्छ						Ad1.8
**36.9**	आफ्नो र आफ्ना बच्चाहरुको स्वास्थ्यका लागि खानपानमा धेरै ध्यान दिनुपर्छ						Ad1.9
**36.10**	आफुले खाने खानेकुरामा पाइने शक्ति (क्यालोरी)को बिचार गर्नुपर्छ						Ad1.10
**36.11**	हल्का पेय पदार्थ (जस्तै : कोकाकोला) गर्मीको समयमा राम्रो हुन्छ						Ad1.11
**36.12**	बच्चाहरुका लागि हल्का पेय पदार्थ जस्तै:कोकाकोला) दिनु ठिक हो किन कि यी पेय पदाथहरु स्वस्थकर हुन्छ						Ad1.12
**36.13**	विमारी पर्नु भनेको आ-आफ्नो भाग्यको कुरा हो, खानेकुराको कारणले हैन						Ad1.13
**36.14**	यदि बच्चाहरु चिन्तित् छन् अथवा खुशी छैनन् भने उनीहरुको मनपर्ने खानाले उनीहरुलाई खुशी पार्दछ						Ad1.14
**36.15**	आफ्नो बच्चालाई माया गरिन्छ भने उनीहरुलाई मनपर्ने मिठाई वा चकलेट किनीदिनु पर्छ						Ad1.15

37. अब म बालबालिकाको स्वास्थ्यमा सञ्चार माध्यमले पार्ने प्रभावका बारेमा केही भनाइहरु पढेर सुनाउनेछु । यी भनाइहरु तपाईको बच्चाहरुको लागि लागु हुन पनि सक्छ वा नहुन पनि सक्छ । यी भनाइहरुमा तपाईको आफ्नो धारणा राख्नुहोला । तपाइ यसमा पूर्ण सहमत वा अलिअलि सहमत वा अलिअलि असहमत वा पूर्ण असहमत वा थाहा छैन भन्न सक्नुहुनेछ ।

(उत्तर मिल्ने कोठामा चिन्ह (√) लाउनुहोला तर प्रत्येक भनाइमा एउटा मात्र उत्तर हुनेछ ।)

**Statements**<br>**भनाईहरु**	**A**<br>**पूर्ण सहमत**	**B**<br>**अलिअलि सहमत**	**C**<br>**अलिअलि असहमत**	**D**<br>**पूर्ण असहमत**	**E**<br>**थाहा छैन**	**कोड**
**37.1** आफ्ना बच्चालाई स्वस्थकर र सन्तुलित खानेकुरा खुवाउने कुरालाई सञ्चार माध्यमको विज्ञापनले अभिभावकलाई मद्दत गरेको हुन्छ ।						Ad2.1
**37.2** सञ्चार माध्यमको विज्ञापनले बालबालिका तथा उनीहरुका अभिभावकलाई अनावश्यक र कहिलेकाहीँ हानीकारक उत्पादन किन्नका लागि उक्साइरहेको हुन्छ।						Ad2.2
**37.3** बालबालिकाको वजन बढ्ने समस्या तथा खराब दाँतका लागि धेरै हदसम्म सञ्चार माध्यमको विज्ञापन जिम्मेवार हुन्छ ।						Ad2.3

**क्र. सं.**	**प्रश्नहरु**	**उत्तर**	**कोड**
**38.**	म तपाईलाई विभिन्न प्रकारका खानेकुराहरु एक-एक गरी पढेर सुनाउनेछु । यीमध्ये कुनकुन खानेकुरा तपाईलाई मिठो वा स्वादिलो लाग्छ ?<br><br>*(दायाँतिर भएका प्रत्येक खानेकुरा पढेर सुनाउनुहोस् र प्रत्येकमा मिठो **लाग्छ** वा **लाग्दैन** भनी सोध्नुहोस् । उत्तरदाताबाट आएको उत्तरलाई सँगैको बाकस भित्र मिठो **लाग्छ** भने **"1"** तथा **लाग्दैन** भने **"0"** र **थाहा छैन** भने **"99"** लेख्नुहोस् ।)*	**प्रश्न** / **उत्तर**<br>1. तारेको तरकारी<br>2. ससेज, पिज्जा<br>3. गुलियो पेय पदार्थ जस्तै कोकाकोला<br>4. फलफुल<br>5. डोनट, बिस्कुट आदि<br>6. चकलेट<br>7. मिठाई<br>8. केक<br>9. आलुचिप्स तथा कुरकुरे<br>10. दाल, भात, तरकारी<br>11. मासु<br>12. झोल भएको चाउचाउ<br>13. सुख्खा चाउचाउ<br>14. रोटी तरकारी<br>15. सलाद (गाजर, मुला, काँक्रा आदि)	Ad3.1<br>Ad3.2<br>Ad3.3<br>Ad3.4<br>Ad3.5<br>Ad3.6<br>Ad3.7<br>Ad3.8<br>Ad3.9<br>Ad3.10<br>Ad3.11<br>Ad3.12<br>Ad3.13<br>Ad3.14<br>Ad3.15
**39.**	खानपिनको सम्बन्धी जानकारी तपाईले को बाट पाउँनुहुन्छ ?<br><br>*(प्राप्त उत्तरमा घेरा लाउनुहोस्)*<br><br>**(बहु-उत्तर प्रश्न)**<br>**(Multiple answer question)**<br><br>*(दायाँतिर भएका उत्तरहरु तुरुन्तै नभन्नुस् । प्रश्न सोधेपछि केही समय पर्खनुस् । यदि सहभागीले कुनैपनि उत्तर भन्न सकेन भने मात्र उक्त उत्तरहरु पढ्नुहोला ।)*	**प्रश्न**<br>1. आफ्नी आमा वा सासू<br>2. अन्य नातेदार<br>3. साथी वा छिमेकी<br>4. टेलिभिजन, रेडियो वा पत्रपत्रिका<br>5. पुस्तकहरु<br>6. विद्युतीय सञ्चार माध्यम ईन्टरनेट<br>7. अन्य (उल्लेख गर्नुहोस् ________<br>8. पोषणसम्बन्धी जानकारी नै छैन<br>9. थाहा छैन वा भन्न सक्दिन	Ad4.1<br>Ad4.2<br>Ad4.4<br>Ad4.5<br>Ad4.6<br>Ad4.7<br>Ad4.8<br>Ad4.9

<table>
<tr><td>40.</td><td>तपाईलाई स्वस्थकर खानेकुराको बारेमा कत्तिको जानकारी छ जस्तो लाग्छ?

(प्राप्त उत्तरमा घेरा लाउनुहोस्)</td><td>1. पर्याप्त
2. ठिकै
3. थोरै वा कम
4. थाहा छैन</td><td>Ad5</td></tr>
<tr><td>41.</td><td>के तपाई स्वस्थकर खानेकुरा सम्बन्धि ज्ञान वा जानकारी पाउँने कोशिस गर्नुहुन्छ ? कसरी ?

(दायाँतिर भएका उत्तरहरु तुरुन्तै नभन्नुस् । प्रश्न सोधेपछि केही समय पर्खनुस् । यदि सहभागीलाई उत्तर भन्न गाह्रो भएमा मात्र उक्त उत्तरहरु पढ्नुहोला ।)</td><td>1. गर्छु , जान्नेलाई सोध्नेछु
2. गर्छु ,त्यसलाई कहीँ कतै पढ्नेछु
3. अँह,यदि चिकित्सकले (डाक्टर) मलाई केही भन्दैनन् भने, त्यो कुरा महत्वपूर्ण हुँदैन
4. अँह, कहाँ खोज्ने वा कस्लाई सोध्ने मलाई थाहा छैन
5. त्यो ज्ञान नभए पनि म मरो खानपानको तौरतरिका आफै व्यवस्थापन गर्छु
6. अँह, मलाई सबै थाहा छ
7. यसको बारेमा पहिले कहिल्यै सोचिन
8. अन्य (उल्लेख गर्ने)__________
9. थाहा छैन वा भन्न चाहन्न</td><td>Ad6</td></tr>
<tr><td>42.</td><td>के तपाई आफ्नो परिवारको खानपानको तौरतरिका सुधार्न चाहनुहुन्छ ?

(प्राप्त उत्तरमा घेरा लाउनुहोस्)</td><td>1. हो, चाहन्छ ____________________
i. यदि चाहनुहुन्छ भने किन ?__________
ii. कस्तो परिवर्तन वा सुधार गर्न चाहनुहुन्छ ?______
2. अँह, चाहन्न ____________________
i. यदि चाहनुहुन्न भने, किन ?__________
3. थाहा छैन वा भन्न सक्दिन______________</td><td>Ad7
Ad7.1.i
Ad7.1.ii
Ad7.2.i</td></tr>
<tr><td>43.</td><td>बढी स्वस्थकर खानेकुरा प्राप्त गर्न वा खानका लागि तपाईलाई के के कुराहरुले प्रभावित गर्छ ?

(दायाँतिर भएका प्रत्येक उत्तर पढेर सुनाउनुहोस् र प्रत्येकमा गर्छवागर्दैनभनी सोध्नुहोस् । उत्तरदाताबाट आएको उत्तरलाई सगैंको बाकसभित्र गर्छ भने “1” तथा गर्दैन भने “0” र थाहा छैन भने “99” लेख्नुहोस् ।)</td><td><table>
<tr><th>प्रश्न</th><th>उत्तर</th></tr>
<tr><td>1. आफ्नै स्वास्थ्य खराब भएमा</td><td></td></tr>
<tr><td>2. स्वास्थ्यकर्मीको सुझाव तथा सरसल्लाह पाएमा</td><td></td></tr>
<tr><td>3. नजिकको नातेदारलाई रोग वा उसको मृत्यु भएमा</td><td></td></tr>
<tr><td>4. आफुसँग धेरै पैसा भएमा</td><td></td></tr>
<tr><td>5. हाम्रो स्थानीय ठाउँमा स्वस्थ खानेकुराहरु पाइने पसलहरु भएमा</td><td></td></tr>
<tr><td>6. परिवारका सदस्यहरुले स्वस्थकर खानेकुरा खाएमा वा खान मानेमा</td><td></td></tr>
<tr><td>7. खानेकुरा वा स्वस्थकर खानपानको राम्रो जानकारी भएमा</td><td></td></tr>
<tr><td>8. अन्य केही भए उल्लेख गर्ने______________</td><td></td></tr>
<tr><td>9. थाहा छैन वा भन्न सक्दिन</td><td></td></tr>
</table></td><td>Ad8.1
Ad8.2
Ad8.3
Ad8.4
Ad8.5
Ad8.6
Ad8.7
Ad8.8
Ad8.9
Ad8.10</td></tr>
</table>

<table>
<tr><td>44.</td><td>बढी स्वस्थकर वा स्वास्थ्यवर्धक खानेकुरा खान वा उपभोग गर्न तपाईलाई यी कारणहरुले समस्या वा अप्ठ्याराहरु हुनसक्छ कि सक्दैन?

(दायाँतिर भएका प्रत्येक उत्तर पढेर सुनाउनुहोस् र प्रत्येकमा हुनसक्छ वा सक्दैनभनी सोध्नुहोस् । उत्तरदाताबाट आएको उत्तरलाई सँगैको बाकसभित्र हुनसक्छ भने "1" सक्दैन भने "0" र थाहा छैन भने "99" लेख्नुहोस् ।)</td><td>

**प्रश्न**	**उत्तर**
1. स्वस्थ खाना खान कुनै समस्या वा अप्ठ्यारो छैन	
2. राम्ररी खाना पकाउनै आउँदैन	
3. स्वस्थकर खानेकुराको बारेमा मलाई त्यति जानकारी नै छैन	
4. व्यस्त जीवनशैली	
5. परिवारका सदस्यहरुको भिन्नभिन्न स्वादको चाहना	
6. आफुलाई मनपर्ने खानेकुरा छोड्न चाहन्न वा सक्दिन	
7. स्वस्थकर खानेकुरा महँगो भएकोले	
8. ईच्छा शक्तिको अभाव	
9. अन्य (उल्लेख गर्ने) ____________	
10. थाहा छैन वा भन्न सक्दिन	

</td><td>Ad9.1
Ad9.2
Ad9.3
Ad9.4
Ad9.5
Ad9.6
Ad9.7
Ad9.8
Ad9.9
Ad9.10</td></tr>
<tr><td>45.</td><td colspan="3">तल राखिएको प्रश्न प्रत्येक (१ देखि ७ वर्ष उमेरका) बच्चाको लागि एक-एक गरी सोध्नुहोला । प्रत्येक बच्चाको उत्तर तल दिइएको बेग्लाबेग्लै कोठामा लेख्नुहोस् ।</td></tr>
<tr><td>बच्चा १</td><td>के तपाई आफ्नो बच्चाको खानपानको तौरतरिका बदल्न वा परिवर्तन गर्न चाहनुहुन्छ ?

(प्राप्त उत्तरमा घेरा लाउनुहोस्)</td><td>1. हो, चाहन्छु
i. यदि चाहनुहुन्छ भने किन ?____________________
____
ii. के के कुरा बदल्न वा परिवर्तन गर्न चाहनुहुन्छ ? _______
____
2. अँह, चाहन्न
i. यदि चाहनुहुन्न भने, किन ?____________________
____
3. थाहा छैन वा भन्न सक्दिन ____________________</td><td>Ad10.1

Ad10.1.1.i
Ad10.1.1.ii

Ad10.1.2.i</td></tr>
<tr><td>बच्चा २</td><td>के तपाई आफ्नो बच्चाको खानपानको तौरतरिका बदल्न वा परिवर्तन गर्न चाहनुहुन्छ ?

(प्राप्त उत्तरमा घेरा लाउनुहोस्)</td><td>1. हो, चाहन्छु
i. यदि चाहनुहुन्छ भने किन ?____________________
____
ii. केके कुरा बदल्न वा परिवर्तन गर्न चाहनुहुन्छ ?_________
____
2. अँह, चाहन्न
i. यदि चाहनुहुन्न भने, किन ?____________________
____
3. थाहा छैन वा भन्न सक्दिन ____________________</td><td>Ad10.2

Ad10.2.1.i
Ad10.2.1.ii

Ad10.2.2.i</td></tr>
<tr><td>बच्चा ३</td><td>के तपाई आफ्नो बच्चाको खानपानको तौरतरिका बदल्न वा परिवर्तन गर्न चाहनुहुन्छ ?

(प्राप्त उत्तरमा घेरा लाउनुहोस्)</td><td>1. हो, चाहन्छु
i. यदि चाहनुहुन्छ भने किन ?____________________
____
ii. केके कुरा बदल्न वा परिवर्तन गर्न चाहनुहुन्छ ?_________
____
2. अँह, चाहन्न
i. यदि चाहनुहुन्न भने, किन ?____________________
____
3. थाहा छैन वा भन्न सक्दिन ____________________</td><td>Ad10.3

Ad10.3.1.i
Ad10.3.1.ii

Ad10.3.2.i</td></tr>
</table>

46.	तपाईको बिचारमा, बच्चाको खानेकुराको छनौटमा के कुराले प्रभाव पार्छ ?<br><br>*(दायाँतिर भएका प्रत्येक उत्तर पढेर सुनाउनुहोस् र प्रत्येकमा **पार्छ** वा **पार्दैन** भनी सोध्नुहोस् । उत्तरदाताबाट आएको उत्तरलाई सँगैको बाकसभित्र **पार्छ** भने **“1” पार्दैन** भने **“0”** र **थाहा छैन** भने **“99”** लेख्नुहोस्।)*	**प्रश्न**	**उत्तर**	
	1. आमाको खानेकुराको चाहना		Ad11.1
	2. परिवारको (आमा बाहेक अन्य) खानेकुराको चाहना		Ad11.2
	3. विद्यालयमा पढाउने कुरा		Ad11.3
	4. विभिन्न विज्ञापन		Ad11.4
	5. बच्चाको साथी-सँगत		Ad11.5
	6. छिमेकीहरु		Ad11.6
	7. कुनै कुराले प्रभाव पार्दैन		Ad11.7
	8. अन्य (उल्लेख गर्ने) ___________		Ad11.8
	9. थाहा छैन वा भन्न सक्दिन		Ad11.9

B. शारीरिक क्रियाकलाप वा अभ्यास

अब म शारीरिक अभ्यास वा क्रियाकलाप प्रति तपाईको बिचार वा धारणा के छ भनी सोध्नेछ ।

47. अब म शारीरिक अभ्यास वा क्रियाकलापका बारेमा केही भनाइहरु पढेर सुनाउनेछु । यी भनाइहरुमा तपाईको आफ्नो धारणा राख्नुहोला । तपाई यसमा पूर्ण सहमत वा अलिअलि सहमत वा अलिअलि असहमत वा पूर्ण असहमत वा थाहा छैन भन्न सक्नुहुनेछ ।
(उत्तर मिल्ने कोठामा चिन्ह (√) लाउनुहोला तर प्रत्येक भनाइमा एउटा मात्र उत्तर हुनेछ ।)

**Statements**<br>**भनाइ**		**A**<br>**पूर्ण सहमत**	**B**<br>**अलिअलि सहमत**	**C**<br>**अलिअलि असहमत**	**D**<br>**पूर्ण असहमत**	**E**<br>**थाहा छैन**	**कोड**
47.1	शारीरिक क्रियाकलाप रमाइलो कार्य हो ।						Ap1.1
47.2	वयश्क-मानिसहरुका लागि शारीरिक क्रियाकलाप महत्वपूर्ण हुँदैन ।						Ap1.2
47.3	कम शारीरिक क्रियाकलाप वा अभ्यास बढी वजन तथा मोटोपनासँग सम्बन्ध राख्दछ ।						Ap1.3
47.4	जसले थोरै खान्छ उसले, शारीरिक रुपमा त्यति सक्रिय भइरहनु आवश्यक छैन ।						Ap1.4
47.5	धेरै शारीरिक क्रियाकलाप वा अभ्यास गर्न हुदैन किन कि यसले भोक पनि बढाउँछ र त्यसपछि तौल पनि बढाउँछ ।						Ap1.5
47.6	मुटुको रोग छ भन्ने पत्ता लागेपछि, आफ्नो शारीरिक अभ्यास रोक्नुपर्छ ।						Ap1.6
47.7	बढी वजनदार व्यक्तिहरु बढी आकर्षक हुन्छन् ।						Ap1.7
47.8	शारीरिक क्रियाकलाप बालबालिकाकास्वास्थ्यका लागि महत्वपूर्ण हुन्छ ।						Ap1.8
47.9	बढी वजन भएका बालबालिकाहरु आकर्षक हुन्छन्						Ap1.9

47.10	बढी वजन भएका बालबालिकाहरु स्वस्थ हुन्छन्						Ap1.10
47.11	बच्चालाई आवश्यक शारीरिक अभ्यास वा क्रियाकलापका लागि विद्यालय पनि जिम्मेवार हुन्छ						Ap1.11
47.12	सुरक्षाको दृष्टिले, बच्चालाई बाहिर खेल्नबाट रोक्नुपर्छ ।						Ap1.12
47.13	आफ्ना बालबालिकाको आवश्यक शारीरिक अभ्यास वा क्रियाकलापका लागि अभिभावकहरु नै मुख्य जिम्मेवार हुन्छन् ।						Ap1.13
47.14	बालबालिकाले प्रयोग गर्ने टेलिभिजन, कम्प्युटर र अन्य विद्युतीय खेलको प्रयोगमा अभिभावकले रोक लगाउँनुपर्छ ।						Ap1.14

अब म तपाईहरुलाई शारीरिक अभ्यास वा क्रियाकलापहरु प्रति तपाईको धारणा सोध्नेछु ।

क्र.सं.	प्रश्नहरु	उत्तर	कोड
**48.**	यदि कुनै ठाउँमा तपाईलाई पुग्नु छ र त्यो ठाउँ २०-२५ मिनेटको पैदल दुरीमा छ भने, तपाई प्राय कसरी जान **मन पराउनुहुन्छ** ?	**1.** साइकलबाट<br>**2.** आफ्नै सवारी साधनबाट<br>**3.** पैदल वा हिंड्दै<br>**4.** बसबाट<br>**5.** त्यति टाढा म जान्न<br>**6.** अन्य (उल्लेख गर्ने) ____________________<br>**7.** थाहा छैन वा भन्न सक्दिन<br>☐	Ap2
**49.**	तपाईको आफ्नो शारीरिक क्रियाकलाप वा अभ्यासलाई कुन **स्तरको मान्नुहुन्छ**?	**1.** शारीरिक रुपमा धेरै सक्रिय<br>**2.** शारीरिक रुपमा ठीकै सक्रिय<br>**3.** शारीरिक रुपमा धेरै सक्रिय मान्दिन<br>**4.** शारीरिक रुपमा सक्रिय मान्दिन<br>**5.** थाहा छैन वा भन्न सक्दिन<br>*यदि उत्तर **1** वा **2** भएमा, प्रश्न नं. **50** मा जाने*<br>*यदि उत्तर **3**, **4** वा **5** भएमा, प्रश्न नं. **51** मा जाने*	Ap3
**50.**	तपाई आफु किन शारीरिक रुपमा सक्रिय हुनुहुन्छ ?<br>(**बहु-उत्तर प्रश्न**)<br>*(दायाँतिर भएका उत्तरहरु तुरुन्तै नभन्नुस् । प्रश्न सोधेपछि केही समय पर्खनुस् । यदि सहभागीले कुनैपनि उत्तर भन्न सकेन भने मात्र उक्त उत्तरहरु पढ्नुहोला ।)*	**प्रश्न** / **उत्तर**<br>**1.** स्वास्थ्य राम्रो राख्न<br>**2.** मेरो दैनिक कर्तव्य वा दैनिकी हो<br>**3.** आफु चुस्तदुरुस्त हुन<br>**4.** आफ्नो पेशा हो<br>**5.** अन्य (उल्लेख गर्ने)____________________<br>**6.** थाहा छैन वा भन्न सक्दिन	Ap4.1<br>Ap4.2<br>Ap4.3<br>Ap4.4<br>Ap4.5<br>Ap4.6

**51.**	तपाई आफ्नो शारीरिक क्रियाकलाप वा अभ्यास बदल्न वा परिवर्तन गर्न चाहनुहुन्छ ?	**1.** हो, चाहन्छु<br>i. यदि चाहनुहुन्छ भने, किन ? ____________________<br>ii. केकस्तो परिवर्तन गर्न चाहनुहुन्छ ? ____________________<br>**2.** अँह, चाहन्न<br>i. यदि चाहनुहुन्न भने, किन ? ____________________<br>**3.** थाहा छैन वा भन्न सक्दिन ____________________	Ap5<br>Ap5.1.i<br>Ap5.1.ii<br>Ap5.2.i<br>Ap5.3
**52.**	शारीरिक अभ्यास वा क्रियाकलापमा अझ बढि सक्रिय हुनका लागि के कस्ता कुराले तपाईलाई अवरोध गर्नसक्छ ?<br>*(दायाँतिर भएका प्रत्येक उत्तर पढेर सुनाउनुहोस् र प्रत्येकले अवरोध गर्नसक्छ वा गर्न सक्दैन भनी सोध्नुहोस् । उत्तरदाताबाट आएको उत्तरलाई सगैंको बाकस भित्र* ***गर्नसक्छ*** *भने* ***"1"गर्न सक्दैन*** *भने* ***"0"*** *र* ***थाहा छैन*** *भने* ***"99"*** *लेख्नुहोस्।)*	see table below	Ap6.1<br>Ap6.2<br>Ap6.3<br>Ap6.4<br>Ap6.5<br>Ap6.6<br>Ap6.7<br>Ap6.8

प्रश्न	उत्तर
**1.** फुर्सदको समय नभएमा	
**2.** बालबच्चा वा बुढाबुढीको स्याहार गर्नुपर्ने भएमा	
**3.** शारीरिक अभ्यास गर्ने मैदान वा ठाउँको अभाव भएमा	
**4.** अन्य कामहरु गर्नुपर्ने भएमा	
**5.** आफ्नो स्वास्थ्यखराब भएमा	
**6.** अल्छी लाग्ने बानीले गर्दा	
**7.** अरुको अगाडि अप्ठ्यारो लाग्ने भएकोले	
**8.** अन्य (उल्लेख गर्ने) ____________________	

**53.**	शारीरिक रुपमा अझ बढि सक्रिय हुन केकस्ता कुराहरु (तत्वहरु) ले तपाईलाई मद्दत गर्छ ?<br>*(दायाँतिर भएका प्रत्येक उत्तर पढेर सुनाउनुहोस् र प्रत्येकले मद्दत गर्छ वा गर्दैन भनी सोध्नुहोस् । उत्तरदाताबाट आएको उत्तरलाई सँगैको बाकस भित्र* ***गर्छ*** *भने* ***"1"गर्दैन*** *भने* ***"0"*** *र* ***थाहा छैन*** *भने* ***"99"*** *लेख्नुहोस्।)*	see table below	Ap7.1<br>Ap7.2<br>Ap7.3<br>Ap7.4<br>Ap7.5<br>Ap7.6<br>Ap7.7

प्रश्न	उत्तर
**1.** आफ्नै स्वास्थ्यखराब भएमा	
**2.** स्वास्थ्यकर्मी को सुझाव वा सल्लाहपाएमा	
**3.** साथी वा नातेदारको सहयोग पाएमा	
**4.** शारीरिक अभ्यास वा क्रियाकलापका बारेमा राम्रो जानकारी भएमा	
**5.** अभ्यासका लागि उपयुक्त ठाउँ भएमा	
**6.** अन्य (उल्लेख गर्ने) ____________________	

अब म तपाईका बालबालिकाहरु (बच्चाहरु)को शारीरिक अभ्यास वा क्रियाकलापहरु प्रति तपाईको धारणा सोध्नेछु ।

यदि सहभागी (उत्तरदाता)को एकभन्दा बढी १ देखि ७ वर्षका बालबालिका छन् भने प्रत्येकलाई बेग्लाबेग्लै नम्बर दिई राख्ने जस्तै १, २, ३ आदि (बढीमा ३ जनाको मात्र) र प्रत्येक बच्चाको जानकारी वा उसकै नाम भएको छेउमा लेख्ने । प्रत्येक प्रश्नको उत्तरमा"√"चिन्ह लगाउनुहोस् ।

**54.**	तपाईको आफ्नो बच्चाको शारीरिक क्रियाकलाप वा अभ्यासलाई कुन स्तरको मान्नुहुन्छ ?<br>*(सहभागी-उत्तरदातालाई आफ्नो १ देखि ७ वर्ष उमेरका प्रत्येक बच्चाको शारीरिक अभ्यासको मुल्यांकन छुट्टा छुट्टै गर्न भन्नुहोस्)*	see table below	Ap8.1<br>Ap8.2<br>Ap8.3

प्रश्न	बच्चा १	बच्चा २	बच्चा ३
1. शारीरिक रुपमा धेरै सक्रिय			
2. शारीरिक रुपमा ठीकै सक्रिय			
3. शारीरिक रुपमा धेरै सक्रिय छैन			
4. शारीरिक रुपमा पटक्कै सक्रिय छैन			
5. थाहा छैन वा भन्न सक्दिन			

<table>
<tr><td>55.</td><td colspan="3">तल राखिएको प्रश्न प्रत्येक (१ देखि ७ वर्ष उमेरका) बच्चाको लागि एक-एक गरी सोध्नुहोला । प्रत्येक बच्चाको उत्तर तल दिइएको बेग्लाबेग्लै कोठामा लेख्नुहोस् ।</td></tr>
<tr><td>बच्चा १</td><td>तपाई आफ्नो बच्चाको शारीरिक क्रियाकलाप वा अभ्यास बदल्न वा परिवर्तन गर्न चाहनुहुन्छ ?</td><td>1. हो, चाहन्छु
i. यदि चाहनुहुन्छ भने, किन ? ____
ii. केकस्तो परिवर्तन गर्न चाहनुहुन्छ ? ____
2. अँह, चाहन्न
i. यदि चाहनुहुन्न भने, किन ? ____
3. थाहा छैन वा भन्न सक्दिन ____</td><td>Ap9.1
Ap9.1.1.i
Ap9.1.1.ii
Ap9.1.2.i
Ap9.3</td></tr>
<tr><td>बच्चा २</td><td>तपाई आफ्नो बच्चाको शारीरिक क्रियाकलाप वा अभ्यास बदल्न वा परिवर्तन गर्न चाहनुहुन्छ ?</td><td>1. हो, चाहन्छु
i. यदि चाहनुहुन्छ भने, किन ? ____
ii. केकस्तो परिवर्तन गर्न चाहनुहुन्छ ? ____
2. अँह, चाहन्न
i. यदि चाहनुहुन्न भने, किन ? ____
3. थाहा छैन वा भन्न सक्दिन ____</td><td>Ap9.1
Ap9.2.2.i
Ap9.2.2.ii
Ap9.2.2.i
Ap9.3</td></tr>
<tr><td>बच्चा ३</td><td>तपाई आफ्नो बच्चाको शारीरिक क्रियाकलाप वा अभ्यास बदल्न वा परिवर्तन गर्न चाहनुहुन्छ ?</td><td>1. हो, चाहन्छु
i. यदि चाहनुहुन्छ भने, किन ? ____
ii. केकस्तो परिवर्तन गर्न चाहनुहुन्छ ? ____
2. अँह, चाहन्न
i. यदि चाहनुहुन्न भने, किन ? ____
3. थाहा छैन वा भन्न सक्दिन____</td><td>Ap9.1
Ap9.3.1.i
Ap9.3.1.ii
Ap9.3.2.i</td></tr>
<tr><td>56.</td><td>शारीरिक रुपमा अझ बढि सक्रिय हुनका लागि के कस्ता कुराले बच्चालाई अवरोध गर्न सक्छ ?
(दायाँतिर भएका प्रत्येक उत्तर पढेर सुनाउनुहोस् र प्रत्येकले अवरोध गर्नसक्छ वा गर्नसक्दैन भनी सोध्नुहोस् । उत्तरदाता बाट आएको उत्तरलाई सँगैको बाकस भित्र <u>गर्नसक्छ</u> भने "1" <u>गर्न सक्दैन</u> भने "0"र <u>थाहा छैन</u> भने "99" लेख्नुहोस्।)</td><td><table><tr><th>प्रश्न</th><th>उत्तर</th></tr><tr><td>1. अति धेरै गृहकार्यभएमा</td><td></td></tr><tr><td>2. घरबाहिर खेल्ने ठाउँको अभावभएमा</td><td></td></tr><tr><td>3. खेल्ने साथी नभएमा</td><td></td></tr><tr><td>4. बच्चालाई बाहिर खेल्न दिनु खतरायुक्त भएमा</td><td></td></tr><tr><td>5. अन्य (उल्लेख गर्ने)____</td><td></td></tr></table></td><td>Ap10.1
Ap10.2
Ap10.3
Ap10.4
Ap10.5</td></tr>
<tr><td>57.</td><td>बच्चालाई शारीरिक रुपमा अझ बढि सक्रिय हुन के कस्ता कुराहरु (तत्वहरु) ले मद्दत गर्छ?
(दायाँतिर भएका प्रत्येक उत्तर पढेर सुनाउनुहोस् र प्रत्येकलेमद्दत गर्छ वा गर्दैन भनी सोध्नुहोस् । उत्तरदाताबाट आएको उत्तरलाई सँगैको बाकसभित्र<u>गर्छ</u> भने "1"<u>गर्दैन</u> भने "0"र <u>थाहा छैन</u> भने "99"लेख्नुहोस्।)</td><td><table><tr><th>प्रश्न</th><th>उत्तर</th></tr><tr><td>1. शारीरिक अभ्यास वा क्रियाकलापलाई अभिभावकले प्रोत्साहन गरेमा</td><td></td></tr><tr><td>2. बाहिर खेल्ने ठाउँ उपलब्ध भएमा</td><td></td></tr><tr><td>3. अभिभावक र बच्चा सँगै खेल्ने गरेमा</td><td></td></tr><tr><td>4. फुर्सदको समय धेरै भएमा</td><td></td></tr><tr><td>5. साथीहरु भएमा</td><td></td></tr><tr><td>6. विद्युत लोडसेडिंगभएमा</td><td></td></tr><tr><td>7. अन्य (उल्लेख गर्ने) ____</td><td></td></tr></table></td><td>Ap11.1
Ap11.2
Ap11.3
Ap11.4
Ap11.5
Ap11.6
Ap11.7</td></tr>
</table>

58.	के तपाई आफ्नो बच्चाको शारीरिक क्रियाकलाप वा अभ्यासमा प्रभाव पार्न सक्नु हुन्छ जस्तो लाग्छ कि लाग्दैन ?	(see table below)	Ap12.1<br>Ap11.2<br>Ap11.3

प्रश्न	बच्चा १	बच्चा २	बच्चा ३
1. सक्छु			
2. सक्दिन			
3. थाहा छैन वा भन्न सक्दिन			

V. आमा तथा बालबालिकाको व्यवहार

A. पारिवारिक खानपानको तौरतरिका

अब म तपाईको परिवारको खानपानको तौरतरिकाको बारेमा सोध्नेछु ।

क्र.सं.	प्रश्नहरु	उत्तर	कोड
59.	तपाईको परिवारमा धेरैजसो कसले खाना पकाउने काम गर्छ ?<br>*(दायाँतिर भएका उत्तरहरु तुरुन्तै नभन्नुस् । प्रश्न सोधेपछि केही समय पर्खनुस् । यदि सहभागीलाई उत्तर भन्न गाह्रो भएमा मात्र उक्त उत्तरहरु पढ्नुहोला ।)*	1. म आफैं<br>2. मेरो श्रीमान् (पति)<br>3. सासू / आमा<br>4. ससूरा / बुबा<br>5. कामगर्ने मान्छे (सहयोगी)<br>6. अन्य (उल्लेख गर्ने) ____________	Bd1
60.	तपाईको परिवारमा खाना के, कस्तो र कसरी पकाउने कुराको निर्णय कसले गर्छ?<br>*(दायाँतिर भएका उत्तरहरु तुरुन्तै नभन्नुस् । प्रश्न सोधेपछि केही समय पर्खनुस् । यदि सहभागीलाई उत्तर भन्न गाह्रो भएमा मात्र उक्त उत्तरहरु पढ्नुहोला ।)*	1. म आफैं<br>2. मेरो श्रीमान् (पति)<br>3. सासू / आमा<br>4. ससूरा / बुबा<br>5. कामगर्ने मान्छे (सहयोगी)<br>6. बालबच्चाले<br>7. हामी सबैले<br>8. अन्य (उल्लेख गर्ने) ____________	Bd2
61.	तपाईको परिवारका हरेक सदस्यहरु सगैं कत्तिको घरबाहिर खाना खाने गर्छन्?<br>*(दायाँतिर भएका उत्तरहरु तुरुन्तै नभन्नुस् । प्रश्न सोधेपछि केही समय पर्खनुस् । यदि सहभागीलाई उत्तर भन्न गाह्रो भएमा मात्र उक्त उत्तरहरु पढ्नुहोला ।)*	1. हरेक दिन<br>2. एक दिन बिराएर<br>3. हप्ता वा साताको एकपटक<br>4. एक महिनामा एकपटक<br>5. कहिल्यै घरबाहिर खाँदैनौं<br>6. अन्य (उल्लेख गर्ने) ____________<br>7. थाहा छैन वा भन्न सक्दिन	Bd3
62.	के तपाईहरु सप्ताहको अन्त्यमा जस्तै शनिबार, अन्य दिनको भन्दा फरक वा बेग्लै तरिकाले खानुहुन्छ? कसरी?<br>*(दायाँतिर भएका उत्तरहरु तुरुन्तै नभन्नुस् । प्रश्न सोधेपछि केही समय पर्खनुस् । यदि सहभागीलाई उत्तर भन्न गाह्रो भएमा मात्र उक्त उत्तरहरु पढ्नुहोला ।)*	1. अरु दिन खाए जस्तै<br>2. विशेष खाना पाक्छ<br>3. नातेदार वा साथीको घरमा खान जान्छौं<br>4. बाहिर (होटल, रेष्टुराँ) खान जान्छौं<br>5. अन्य (उल्लेख गर्ने) ____________<br>6. थाहा छैन वा भन्न सक्दिन	Bd4

**63.**	तपाईको घरमा, खाना खाने समयमा गुलियो पेयपदार्थ जस्तै कोकाकोला पनि खाने गरिन्छ?<br>*(दायाँतिर भएका उत्तरहरु तुरुन्तै नभन्नुस् । प्रश्न सोधेपछि केही समय पर्खनुस् । यदि सहभागीलाई उत्तर भन्न गाह्रो भएमा मात्र उक्त उत्तरहरु पढ्नुहोला ।)*	1. हो, प्रायजसो हुन्छ<br>2. कहिलेकाहीँ<br>3. प्रायजसो हुँदैन<br>4. थाहा छैन वा भन्न सक्दिन	Bd5
**64.**	के तपाई आफ्नो घरमा जंक फुड नियन्त्रण गर्नुहुन्छ ?<br>*(दायाँतिर भएका उत्तरहरु तुरुन्तै नभन्नुस् । प्रश्न सोधेपछि केही समय पर्खनुस् । यदि सहभागीलाई उत्तर भन्न गाह्रो भएमा मात्र उक्त उत्तरहरु पढ्नुहोला ।)*	1. बजारु खानेकुरा वा जंक फुड भनेको थाहा छैन<br>2. घरमा स्वस्थकर खानेकुरा राख्ने कोशिस गर्छु तर पनि केही अस्वस्थकर खानेकुरा भइहाल्छ ।<br>3. परिवारमा केकस्तो खानेकुरा दिइन्छ वास्ता नै हुँदैन<br>4. घरमा स्वस्थकर खानेकुरा मात्र हुन्छ<br>5. अन्य (उल्लेख गर्ने) ______________<br>6. थाहा छैन वा भन्न सक्दिन	Bd6

***B.* बच्चाको खापपानको तौरतरिका**

**अब म तपाईका बालबालिकाहरु (बच्चाहरु)को खानपानको तौरतरिकाको बारेमा सोध्नेछु ।**			
यदि सहभागी (उत्तरदाता)को एकभन्दा बढी १ देखि ७ वर्षका बालबालिका छन् भने प्रत्येकलाई बेग्लाबेग्लै नम्बर दिई राख्ने जस्तै १, २, ३ आदि (बढीमा ३ जनाको मात्र) र प्रत्येक बच्चाको जानकारी वा उसकै नाम भएको छेउमा लेख्ने । प्रत्येक प्रश्नको उत्तरमा "√" चिन्ह लगाउनुहोस् ।			
**क्र.सं.**	**प्रश्नहरु**	**उत्तर**	**कोड**
**65.**	तल राखिएको प्रश्न प्रत्येक (१ देखि ७ वर्ष उमेरका) बच्चाको लागि एक-एक गरी सोध्नुहोला । प्रत्येक बच्चाको उत्तर तल दिइएको बेग्लाबेग्लै कोठामा लेख्नुहोस् ।		
**बच्चा १**	के तपाई प्रायजसो आफुले खाने गरेको खानेकुरा नै आफ्ना बच्चा (बच्चाहरु) लाई खान दिनुहुन्छ ?<br>*(प्राप्त उत्तरमा घेरा लाउनुहोस्)*	1. हो, त्यस्तै दिन्छौं<br>2. अँह, त्यस्तै दिन्नौं<br>i. यदि दिनुहुन्न भने किन ?<br>______________<br>3. अन्य (उल्लेख गर्ने) ______________<br>4. थाहा छैन वा भन्न सक्दिन	Bd7.1<br>Bd7.1.2.i
**बच्चा २**	के तपाई प्रायजसो आफुले खाने गरेको खानेकुरा नै आफ्ना बच्चा (बच्चाहरु) लाई खान दिनुहुन्छ ?<br>*(प्राप्त उत्तरमा घेरा लाउनुहोस्)*	1. हो, त्यस्तै दिन्छौं<br>2. अँह, त्यस्तै दिन्नौं<br>i. यदि दिनुहुन्न भने किन ?<br>______________<br>3. अन्य (उल्लेख गर्ने) ______________<br>4. थाहा छैन वा भन्न सक्दिन	Bd7.2<br>Bd7.2.2.i
**बच्चा ३**	के तपाई प्रायजसो आफुले खाने गरेको खानेकुरा नै आफ्ना बच्चा (बच्चाहरु) लाई खान दिनुहुन्छ ?<br>*(प्राप्त उत्तरमा घेरा लाउनुहोस्)*	1. हो, त्यस्तै दिन्छौं<br>2. अँह, त्यस्तै दिन्नौं<br>i.यदि दिनुहुन्न भने किन ?______________<br>3. अन्य (उल्लेख गर्ने) ______________<br>4. थाहा छैन वा भन्न सक्दिन	Bd7.3<br>Bd7.3.2.i

66.

तपाईको बच्चा (बच्चाहरु) ले विद्यालयको टिफीन समयमा के खानेकुरा खान्छ ?

(दायाँतिर भएका उत्तरहरु तुरुन्तै नभन्नुस् । प्रश्न सोधेपछि केही समय पर्खनुस् । यदि सहभागीलाई उत्तर भन्न गाह्रो भएमा मात्र उक्त उत्तरहरु पढ्नुहोला ।)

**प्रश्न**	**बच्चा १**	**बच्चा २**	**बच्चा ३**
1. सँधै घरबाट लगेको खाजा खान्छ			
2. कहिलेकाहीँ विद्यालयले नै दिएको खान्छ वा त्यहीँ किनेर खान्छ , कहिलेकाहीँ घरबाट लिएर जान्छ			
3. सँधै विद्यालयले दिने खाजा खाने गर्छ			
4. नजिकको पसलबाट खाजा किनेर खाने			
5. अन्य (उल्लेख गर्ने) ______________			
6. विद्यालय नै जाँदैन			
7. थाहा छैन वा भन्न सक्दिन			

Bd8.1
Bd8.2
Bd8.3

67.

तपाईको बच्चा (बच्चाहरु)ले कुन ठाउँमा सबैभन्दा धेरै स्वस्थकर वा स्वास्थ्यबर्धक खानेकुरा खान्छ ?

(दायाँतिर भएका उत्तरहरु तुरुन्तै नभन्नुस् । प्रश्न सोधेपछि केही समय पर्खनुस् । यदि सहभागीलाई उत्तर भन्न गाह्रो भएमा मात्र उक्त उत्तरहरु पढ्नुहोला ।)

**प्रश्न**	**बच्चा १**	**बच्चा २**	**बच्चा ३**
1.घरमा			
2.विद्यालयमा			
3.घरबाहिर (होटल आदि)मा			
4.अन्य (उल्लेख गर्ने) ______________			
5.थाहा छैन वा भन्न सक्दिन			

Bd9.1
Bd9.2
Bd9.3

68.

तपाईको बच्चा (बच्चाहरु)ले कति कति समयमा गोजीखर्च वा पाकेट खर्च लाने गर्छ?

(दायाँतिर भएका उत्तरहरु तुरुन्तै नभन्नुस् । प्रश्न सोधेपछि केही समय पर्खनुस् । यदि सहभागीलाई उत्तर भन्न गाह्रो भएमा मात्र उक्त उत्तरहरु पढ्नुहोला ।)

**प्रश्न**	**बच्चा १**	**बच्चा २**	**बच्चा ३**
1. सँधै वा प्रत्येक दिन			
2. सातामा दुई-तीन पटक			
3. सातामा एकपटक			
4. कहिले पनि लाँदैन			
5. अन्य (उल्लेख गर्ने) ______________			
6. थाहा छैन वा भन्न सक्दिन			

*यदि कुनै पनि नम्बरको बच्चाको लागि उत्तर **4** वा **6** आउँछ भने, प्रश्न नं. **70** मा त्यही नम्बरको बच्चाको कोठामा जाने । यदि, अरु बच्चाको लागि उत्तर नं. **4** वा **6** छैन भने उनीहरुको शुरुवात प्रश्न नं. **69** बाटनै गर्ने ।*

Bd10.1
Bd10.2
Bd10.3

69.	यदि तपाईको बच्चा (बच्चाहरु)ले गोजीखर्च (पाकेट खर्च) लान्छ भने, तिनीहरुले त्यो पैसा केका लागि कसरी खर्च गर्छन् ?<br><br>**(बहु-उत्तर प्रश्न)**<br>**(Multiple answer question)**<br>*(दायाँतिर भएका उत्तरहरु तुरुन्तै नभन्नुस् । प्रश्न सोधेपछि केही समय पर्खनुस् । यदि सहभागीलाई उत्तर भन्न गाह्रो भएमा मात्र उक्त उत्तरहरु पढ्नुहोला ।)*	(see table below)	Child 1:<br>Bd11.1.1<br>Bd11.1.2<br>Bd11.1.3<br>Bd11.1.4<br>Bd11.1.5<br>Bd11.1.6<br>Bd11.1.7<br>(For child 2and 3 – similar format:<br>Bd11.2.1 etc)

प्रश्न	बच्चा १	बच्चा २	बच्चा ३
**1.**खानेकुरा किन्छ			
**2.**चकलेट किन्छ			
**3.**पेयपदार्थ जस्तै कोकाकोला किन्छ			
**4.**खेल्ने चिज जस्तै गुडिया आदि किन्छ			
**5.**इन्टरनेटमा खेल (गेम) खेल्छ			
**6.**बचत गर्छ			
**7.**अन्य (उल्लेख गर्ने) ____________________			
**8.**म वास्तै गर्दिन			
**9.**थाहा छैन वा भन्न सक्दिन			

70. तपाईलाई म विभिन्न प्रकारका खानेकुराहरु एकएक गरी भन्छु र तपाईले मलाई भन्नुस् कि यीमध्ये कुन चाहीँ खानेकुरा तपाईको बालबच्चाले खाजाको रुपमा खाने गर्छ ? सम्भावित उत्तर प्रायजसो वा कहिलेकाहीँ वा प्राय खाँदैनन् वा थाहा छैन हुन् ।
(ठीक उत्तर आएको कोठामा (√) चिन्ह प्रयोग गर्नुस् । प्रत्येक भनाइमा एउटा मात्र ठीक उत्तर छान्नुहोस् ।)

बच्चा १

**Items**<br>**खानेकुराको प्रकार**	**A**<br>**प्रायजसो**	**B**<br>**कहिलेकाहीँ**	**C**<br>**प्राय खाँदैनन्**	**D**<br>**थाहा छैन**	**कोड**
**70.1.** चाउमिन					Bd12.1.1
**70.2.** तयारी चाउचाउ					Bd12.1.2
**70.3.** घरमै बनाएको रोटी तरकारी					Bd12.1.3
**70.4.** मासु वा तरकारी तथा च्युरा					Bd12.1.4
**70.5.** बिस्कुट तथा पाउरोटी					Bd12.1.5
**70.6.** मिठाई					Bd12.1.6
**70.7.** चटपटे, समोसा, पानीपुरी					Bd12.1.7
**70.8.** मोःमोः					Bd12.1.8
**70.9.** अण्डा					Bd12.1.9
**70.10.** दुध					Bd12.1.10
**70.11.** चिया					Bd12.1.11
**70.12.** दही					Bd12.1.12
**70.13.** ताजा सलाद (गाजर, काँक्रा, मुला)					Bd12.1.13
**70.14.** भुटेको / उसिनेको मकै					Bd12.1.14
**70.15.** फलफुल					Bd12.1.15
**70.16.** अन्य (उल्लेख गर्ने)					Bd12.1.16

बच्चा २

Items<br>खानेकुराको प्रकार	A<br>प्रायजसो	B<br>कहिलेकाहीँ	C<br>प्राय खाँदैनन्	D<br>थाहा छैन	कोड
**70.1.** चाउमिन					Bd12.2.1
**70.2.** तयारी चाउचाउ					Bd12.2.2
**70.3.** घरमै बनाएको रोटी तरकारी					Bd12.2.3
**70.4.** मासु वा तरकारी तथा च्युरा					Bd12.2.4
**70.5.** बिस्कुट तथा पाउरोटी					Bd12.2.5
**70.6.** मिठाई					Bd12.2.6
**70.7.** चटपटे, समोसा, पानीपुरी					Bd12.2.7
**70.8.** मोःमोः					Bd12.2.8
**70.9.** अण्डा					Bd12.2.9
**70.10.** दुध					Bd12.2.10
**70.11.** चिया					Bd12.2.11
**70.12.** दही					Bd12.2.12
**70.13.** ताजा सलाद (गाजर, काँक्रा, मुला)					Bd12.2.13
**70.14.** भुटेको / उसिनेको मकै					Bd12.2.14
**70.15.** फलफुल					Bd12.2.14
**70.16.** अन्य (उल्लेख गर्ने)					Bd12.2.15

बच्चा ३

Items<br>खानेकुराको प्रकार	A<br>प्रायजसो	B<br>कहिलेकाहीँ	C<br>प्राय खाँदैनन्	D<br>थाहा छैन	कोड
**70.1.** चाउमिन					Bd12.3.1
**70.2.** तयारी चाउचाउ					Bd12.3.2
**70.3.** घरमै बनाएको रोटी तरकारी					Bd12.3.3
**70.4.** मासु वा तरकारी तथा च्युरा					Bd12.3.4
**70.5.** बिस्कुट तथा पाउरोटी					Bd12.3.5
**70.6.** मिठाई					Bd12.3.6
**70.7.** चटपटे, समोसा, पानीपुरी					Bd12.3.7
**70.8.** मोःमोः					Bd12.3.8
**70.9.** अण्डा					Bd12.3.9
**70.10.** दुध					Bd12.3.10
**70.11.** चिया					Bd12.3.11
**70.12.** दही					Bd12.3.12
**70.13.** ताजा सलाद (गाजर, काँक्रा, मुला)					Bd12.3.13
**70.14.** भुटेको / उसिनेको मकै					Bd12.3.14
**70.15.** फलफुल					Bd12.3.15
**70.16.** अन्य (उल्लेख गर्ने)					Bd12.3.16

क्र.सं.	प्रश्नहरु	उत्तर	कोड
71.	**प्रत्येक (१ देखि ७ वर्षसम्मका) बच्चाका लागि बेग्लाबेग्लै ठाउँको प्रयोग गर्नुहोस् ।**		
**बच्चा १**	तपाईको बच्चाले बढी मनपराउने कुनै तीनवटा खानेकुरा केके हुन् ?	1. ____________<br>2. ____________<br>3. ____________<br>4. थाहा छैन वा भन्न सक्दिन	Bd13.1.1<br>Bd13.1.2<br>Bd13.1.3<br>Bd13.1.4
**बच्चा २**	तपाईको बच्चाले बढी मनपराउने कुनै तीनवटा खानेकुरा केके हुन् ?	1. ____________<br>2. ____________<br>3. ____________<br>4. थाहा छैन वा भन्न सक्दिन	Bd13.2.1<br>Bd13.2.2<br>Bd13.2.3<br>Bd13.2.4
**बच्चा ३**	तपाईको बच्चाले बढी मनपराउने कुनै तीनवटा खानेकुरा केके हुन् ?	1. ____________<br>2. ____________<br>3. ____________<br>4. थाहा छैन वा भन्न सक्दिन	Bd13.3.1<br>Bd13.3.2<br>Bd13.3.3<br>Bd13.3.4

72. तपाईको बच्चाले बढी मनपराउने पेयपदार्थ (पिउने कुरा) के हो ?
(बहु-उत्तर प्रश्न)
(Multiple answer question)
(दायाँतिर भएका उत्तरहरु तुरुन्तै नभन्नुस् । प्रश्न सोधेपछि केही समय पर्खनुस् । यदि सहभागीलाई उत्तर भन्न गाह्रो भएमा मात्र उक्त उत्तरहरु पढ्नुहोला ।)

प्रश्न	बच्चा १	बच्चा २	बच्चा ३
1. पानी			
2. दुध			
3. चिया			
4. पाकेटको जुस जस्तै रियल जुस, स्लाइस आदि			
5. पेय पदार्थ जस्तै कोकाकोला आदि			
6. फलफुलको ताजा रस			
7. हर्लिक्स, बुस्ट, कम्प्लान			
8. ट्यांग			
9. ग्लुकोज पानी			
10. अन्य (उल्लेख गर्ने)______			
11. थाहा छैन वा भन्न सक्दिन			

Child 1:
Bd14.1.1
Bd14.1.2
Bd14.1.3
Bd14.1.4
Bd14.1.5
Bd14.1.6
Bd14.1.7
Bd14.1.8
Bd14.1.9
Bd14.1.10
(For child 2and 3 – similar format:

Bd14.2.1 etc)

73. तपाईको बच्चाले कत्तिको (कति पटक) हल्का पेयपदार्थ जस्तै कोकाकोला पिउने गर्छ ?

प्रश्न	बच्चा १	बच्चा २	बच्चा ३
1. दिनमा दुई पटक			
2. दिनको एकपटक			
3. सातामा एकपटक			
4. सातामा दुई पटक			
5. कहिले पनि पिउँदैन			
6. अन्य (उल्लेख गर्ने) ____________			
7. थाहा छैन वा भन्न सक्दिन			

Bd15.1
Bd15.2
Bd15.3

<table>
<tr><td>74.</td><td colspan="3">प्रत्येक (१ देखि ७ वर्षसम्मका) बच्चाका लागि बेग्लाबेग्लै ठाउँको प्रयोग गर्नुहोस् ।</td></tr>
<tr><td>बच्चा १</td><td>के तपाईको बच्चाले टेलिभिजन हेरिरहेको समयमा वा कम्प्युटरमा खेलिरहेको समयमा पनि खाने-पिउने गर्छ ?
(प्राप्त उत्तरमा घेरा लाउनुहोस्)</td><td>1. हो, खान्छ ।
i. यदि खान्छ भने, कति पटक …… (दिन वा हप्तामा)
2. अँह, खाँदैन
3. थाहा छैन वा भन्न सक्दिन
(यदि खाँदैन भने, प्रश्न नं. 78 मा जाने)</td><td>Bd16.1
Bd16.1.1.i</td></tr>
<tr><td>बच्चा २</td><td>के तपाईको बच्चाले टेलिभिजन हेरिरहेको समयमा वा कम्प्युटरमा खेलिरहेको समयमा पनि खाने-पिउन गर्छ ?
(प्राप्त उत्तरमा घेरा लाउनुहोस्)</td><td>1. हो, खान्छ ।
i. यदि खान्छ भने, कति पटक …… (दिन वा हप्तामा)
2. अँह, खाँदैन
3. थाहा छैन वा भन्न सक्दिन
(यदि खाँदैन भने, प्रश्न नं. 78 मा जाने)</td><td>Bd16.2
Bd16.2.1.i</td></tr>
<tr><td>बच्चा ३</td><td>के तपाईको बच्चाले टेलिभिजन हेरिरहेको समयमा वा कम्प्युटरमा खेलिरहेको समयमा पनि खाने-पिउन गर्छ ?
(प्राप्त उत्तरमा घेरा लाउनुहोस्)</td><td>1. हो, खान्छ ।
i. यदि खान्छ भने, कति पटक …… (दिन वा हप्तामा)
2. अँह, खाँदैन
3. थाहा छैन वा भन्न सक्दिन
(यदि खाँदैन भने, प्रश्न नं. 78 मा जाने)</td><td>Bd16.3
Bd16.3.1.i</td></tr>
<tr><td>75.</td><td>यदि तपाईको बच्चाले टेलिभिजन हेरिरहेको वा कम्प्युटरमा खेलिरहेको समयमा पनि खाने-पिउने गर्छ भने, के खाने वा पिउनेकुरा खान्छ/पिउँछ ?

(Multiple answer qurstion)
(बहु-उत्तर प्रश्न)

(दायाँतिर भएका उत्तरहरु तुरुन्तै नभन्नुस् । प्रश्न सोधेपछि केही समय पर्खनुस् । यदि सहभागीलाई उत्तर भन्न गाह्रो भएमा मात्र उक्त उत्तरहरु पढ्नुहोला ।)</td><td>
<table>
<tr><th>प्रश्न</th><th>बच्चा १</th><th>बच्चा २</th><th>बच्चा ३</th></tr>
<tr><td>1. फलफुल</td><td></td><td></td><td></td></tr>
<tr><td>2. कुकिज, विस्कुट</td><td></td><td></td><td></td></tr>
<tr><td>3. आलु चिप्स, कुरकुरे, तयारी चाउचाउ</td><td></td><td></td><td></td></tr>
<tr><td>4. चकलेट</td><td></td><td></td><td></td></tr>
<tr><td>5. सुख्खा फलफुल, बदाम, गेडागुडी</td><td></td><td></td><td></td></tr>
<tr><td>6. हल्का पेयपदार्थ जस्तै कोकाकोला</td><td></td><td></td><td></td></tr>
<tr><td>7. खाना खाने समयमा मात्र</td><td></td><td></td><td></td></tr>
<tr><td>8. अन्य (उल्लेख गर्ने) ______</td><td></td><td></td><td></td></tr>
<tr><td>9. थाहा छैन वा भन्न सक्दिन</td><td></td><td></td><td></td></tr>
</table>
</td><td><u>Child 1:</u>
Bd17.1.1
Bd17.1.2
Bd17.1.3
Bd17.1.4
Bd17.1.5
Bd17.1.6
Bd17.1.7
Bd17.1.8

(For child 2and 3 – similar format: Bd17.2.1 etc)</td></tr>
<tr><td>76.</td><td>के तपाईको बच्चाले टेलिभिजनमा देखेको खानेकुरा ल्याइदिनु (किनिदिनु) भन्छ ?
(दायाँतिर भएका उत्तरहरु तुरुन्तै नभन्नुस् । प्रश्न सोधेपछि केही समय पर्खनुस् । यदि सहभागीलाई उत्तर भन्न गाह्रो भएमा मात्र उक्त उत्तरहरु पढ्नुहोला ।)</td><td>
<table>
<tr><th>प्रश्न</th><th>बच्चा १</th><th>बच्चा २</th><th>बच्चा ३</th></tr>
<tr><td>1. अँह, भन्दैन</td><td></td><td></td><td></td></tr>
<tr><td>2. कहिलेकाहीँ भन्छ</td><td></td><td></td><td></td></tr>
<tr><td>3. प्रायजसो भन्छ</td><td></td><td></td><td></td></tr>
<tr><td>4. मेरो बच्चाले खासै टेलिभिजन नै हेर्दैन</td><td></td><td></td><td></td></tr>
<tr><td>5. थाहा छैन वा भन्न सक्दिन</td><td></td><td></td><td></td></tr>
</table>
</td><td>Bd18.1
Bd18.2
Bd18.3</td></tr>
</table>

<table>
<tr>
<td>77.</td>
<td>तपाई बजार वा पसल गएको बेला, तपाईको बच्चाले कुनै खानेकुरा किनिदिनु भनेको छ जुन पहिले तपाईले किन्छु भनेर सोच्नुभएको थिएन तर के, पछि किनिदिनु हुन्छ?
(प्रत्येक बच्चाको लागि बेग्लाबेग्लै सोध्ने)
(दायाँतिर भएका उत्तरहरु तुरुन्तै नभन्नुस् । प्रश्न सोधेपछि केही समय पर्खनुस् । यदि सहभागीलाई उत्तर भन्न गाह्रो भएमा मात्र उक्त उत्तरहरु पढ्नुहोला ।)</td>
<td>
<table>
<tr><th>प्रश्न</th><th>बच्चा १</th><th>बच्चा २</th><th>बच्चा ३</th></tr>
<tr><td>1. अँह, प्राय किनिदिन्न</td><td></td><td></td><td></td></tr>
<tr><td>2. कहिलेकाहीँ</td><td></td><td></td><td></td></tr>
<tr><td>3. प्रायजसो</td><td></td><td></td><td></td></tr>
<tr><td>4. मसँग पैसा भए किनिदिन्छु</td><td></td><td></td><td></td></tr>
<tr><td>5. उक्त खानेकुरा स्वस्थकर छभने मात्र किनिदिन्छु</td><td></td><td></td><td></td></tr>
<tr><td>6. अन्य (उल्लेख गर्ने) ________________</td><td></td><td></td><td></td></tr>
<tr><td>7. थाहा छैन वा भन्न सक्दिन</td><td></td><td></td><td></td></tr>
</table>
</td>
<td>Bd19.1
Bd19.2
Bd19.3</td>
</tr>
<tr>
<td>78.</td>
<td>तपाईको बच्चाले सँधैजसो बिहानको मुख्य खाना कहाँ खाने गर्छ ?
(दायाँतिर भएका उत्तरहरु तुरुन्तै नभन्नुस् । प्रश्न सोधेपछि केही समय पर्खनुस् । यदि सहभागीलाई उत्तर भन्न गाह्रो भएमा मात्र उक्त उत्तरहरु पढ्नुहोला ।)</td>
<td>
<table>
<tr><th>प्रश्न</th><th>बच्चा १</th><th>बच्चा २</th><th>बच्चा ३</th></tr>
<tr><td>1. घरमा</td><td></td><td></td><td></td></tr>
<tr><td>2. विद्यालयमा</td><td></td><td></td><td></td></tr>
<tr><td>3. विद्यालय जाँदा बाटो छेउ पसलमा</td><td></td><td></td><td></td></tr>
<tr><td>4. अन्य (उल्लेख गर्ने) ________________</td><td></td><td></td><td></td></tr>
<tr><td>5. थाहा छैन वा भन्न सक्दिन</td><td></td><td></td><td></td></tr>
</table>
</td>
<td>Bd20.1
Bd20.2
Bd20.3</td>
</tr>
<tr>
<td>79.</td>
<td colspan="3">प्रत्येक (१ देखि ७ वर्षसम्मका) बच्चाका लागि बेग्लाबेग्लै ठाउँको प्रयोग गर्नुहोस् ।</td>
</tr>
<tr>
<td>बच्चा १</td>
<td>तपाईको बच्चाले बिहानको खानामा सँधैजसो के खाने गर्छ ?</td>
<td>1. ________________________
2. ________________________
3. ________________________</td>
<td>Bd21.1.1
Bd21.1.2
Bd21.1.3</td>
</tr>
<tr>
<td>बच्चा २</td>
<td>तपाईको बच्चाले बिहानको खानामा सँधैजसो के खाने गर्छ ?</td>
<td>1. ________________________
2. ________________________
3. ________________________</td>
<td>Bd21.2.1
Bd21.2.2
Bd21.2.3</td>
</tr>
<tr>
<td>बच्चा ३</td>
<td>तपाईको बच्चाले बिहानको खानामा सँधैजसो के खाने गर्छ ?</td>
<td>1. ________________________
2. ________________________
3. ________________________</td>
<td>Bd21.3.1
Bd21.3.2
Bd21.3.3</td>
</tr>
<tr>
<td>80.</td>
<td colspan="3">प्रत्येक (१ देखि ७ वर्षसम्मका) बच्चाका लागि बेग्लाबेग्लै ठाउँको प्रयोग गर्नुहोस् ।</td>
</tr>
<tr>
<td>बच्चा १</td>
<td>तपाईको बच्चाले रातिको खानामा सँधैजसो के खाने गर्छ ?</td>
<td>1. ________________________
2. ________________________</td>
<td>Bd22.1.1
Bd22.1.2</td>
</tr>
<tr>
<td>बच्चा २</td>
<td>तपाईको बच्चाले रातिको खानामा सँधैजसो के खाने गर्छ ?</td>
<td>1. ________________________
2. ________________________</td>
<td>Bd22.2.1
Bd22.2.2</td>
</tr>
<tr>
<td>बच्चा ३</td>
<td>तपाईको बच्चाले रातिको खानामा सँधैजसो के खाने गर्छ ?</td>
<td>1. ________________________
2. ________________________</td>
<td>Bd22.3.1
Bd22.3.2</td>
</tr>
</table>

C. *Physical activity* शारीरिक क्रियाकलाप वा अभ्यास

अब म तपाईको शारीरिक क्रियाकलाप वा अभ्यास केकस्तो छ , सोध्ने छु ।

81. तपाईले प्राय दिनैजसो कामको समयमा तथा फुसर्दको समयमा **बढी परिश्रम पर्ने** शारीरिक क्रियाकलाप बारेमा छुट्टाछुट्टै कुरा गरौं ।

**81.1 Work कामको समयमा बढी परिश्रम पर्ने शारीरिक क्रियाकलाप**<br>(भारी उचाल्ने, पानी बोक्ने, खेतबारी खन्ने, वा निर्माणको काम आदि)	**Code**
तपाईले **कामको समयमा** बढी परिश्रम पर्ने शारीरिक क्रियाकलापको बारेमा भन्नुस् । तपाईले **बढी परिश्रम लगाएर** लगातार कम्तीमा १० मिनेटसम्म कुनै कार्य वा गतिविधी वा क्रियाकलाप गर्नुहुन्छ जसले गर्दा तपाईको **श्वास** बढ्छ ?<br>*यदि काम गर्नुहुन्छ भने "Yes" मा घेरा लाउनुस् र प्रश्न नं.* ***81.1a*** *मा जानुहोस् । यदि "No" हो भने प्रश्न नं.* ***81.2*** *मा जानुहोस्।*	

**Yes** यदि, गर्नुहुन्छ भने		**No** गर्नुहुन्न भने ***81.2*** मा जाने	Code
81.1a हप्तामा कति दिन गर्नुहुन्छ ?	.... दिन		Bp1.1<br>Bp1.1.a<br>Bp1.1.b
81.1b **प्रतिदिन कति समयसम्म गर्नुहुन्छ ?**	..........घण्टा......... मिनेट		

**81.2 Leisure time फुर्सदको समयमा बढी परिश्रम पर्ने शारीरिक क्रियाकलाप**<br>(दौडने वा शरीर थकित हुने खेल, गह्रौं बस्तु उचाल्ने आदि)	**Code**
तपाईले **फुर्सदको समयमा** बढी परिश्रम पर्ने शारीरिक क्रियाकलापको बारेमा भन्नुस् । तपाईले **बढी परिश्रम लगाएर** लगातार कम्तीमा १० मिनेटसम्म कुनै कार्य वा गतिविधी वा क्रियाकलाप गर्नुहुन्छ जसले गर्दा तपाईको **श्वास** बढ्छ ?<br>*यदि काम गर्नुहुन्छ भने "Yes" मा घेरा लाउनुस् र प्रश्न नं.* ***81.2a*** *मा जानुहोस् । यदि "No" हो भने प्रश्न नं.* ***82*** *मा जानुहोस् ।*	

**Yes** यदि, गर्नुहुन्छ भने।		**No** गर्नुहुन्न भने ***82*** मा जाने	Code
81.2a हप्तामा कति दिन गर्नुहुन्छ ?	.... दिन		Bp1.2<br>Bp1.2.a<br>Bp1.2.b
81.2b **प्रतिदिन कति समयसम्म गर्नुहुन्छ ?**	..........घण्टा......... मिनेट		

82. तपाईले प्राय दिनैजसो कामको समयमा तथा फुसर्दको समयमा **मध्यम परिश्रम पर्ने** शारीरिक क्रियाकलाप बारेमा छुट्टाछुट्टै कुरा गरौं ।

**82.1 Work कामको समयमा मध्यम परिश्रम पर्ने शारीरिक क्रियाकलाप**<br>लुगा धुने, दुध दुहने, गाई बस्तु चऱ्हाउने, भुईं लिप्ने पुच्छने, बाली सुकाउने, हलुका भारी बोक्ने आदि)	**Code**
तपाईले **कामको समयमा** मध्यम परिश्रम पर्ने शारीरिक क्रियाकलापको बारेमा भन्नुस् । तपाईले **मध्यम परिश्रम लगाएर** लगातार कम्तीमा १० मिनेटसम्म कुनै कार्य वा गतिविधी वा क्रियाकलाप गर्नुहुन्छ जसले गर्दा तपाईको श्वास **अलिअलि मात्र** बढ्छ ?<br>*यदि काम गर्नुहुन्छ भने "Yes" मा घेरा लाउनुस् र प्रश्न नं.* ***82.1a*** *मा जानुहोस् । यदि "No" हो भने प्रश्न नं.* ***82.2*** *मा जानुहोस्।*	

**Yes** यदि, गर्नुहुन्छ भने।		**No** गर्नुहुन्न भने ***82.2*** मा जाने	Code
82.1a हप्तामा कति दिन गर्नुहुन्छ ?	.... दिन		Bp2.1<br>Bp2.1.a<br>Bp2.1.b
82.1b **प्रतिदिन कति समयसम्म गर्नुहुन्छ ?**	..........घण्टा......... मिनेट		

**82.2 Leisure time फुर्सदको समयमा मध्यम परिश्रम पर्ने शारीरिक क्रियाकलाप**<br>(फटाफट हिंड्ने, साइकल कुदाउने, सक्रिय योगा, नाच्ने)			**Code**
तपाईले **फुर्सदको समयमा** मध्यम परिश्रम पर्ने शारीरिक क्रियाकलापको बारेमा भन्नुस् । तपाईले **मध्यम परिश्रम लगाएर** लगातार कम्तीमा १० मिनेटसम्म कुनै कार्य वा गतिविधी वा क्रियाकलाप गर्नुहुन्छ जसले गर्दा तपाईको श्वास **अलिअलि मात्र** बढ्छ ?<br>*यदि काम गर्नुहुन्छ भने "Yes" मा घेरा लाउनुस् र प्रश्न नं.* ***82.2a*** *मा जानुहोस् । यदि "No" हो भने प्रश्न नं.**83** मा जानुहोस्।*			
**Yes** यदि, गर्नुहुन्छ भने।		**No** गर्नुहुन्न भने ***83*** मा जाने	Bp2.2<br>Bp2.2.a<br>Bp2.2.b
82.2a हप्तामा कति दिन गर्नुहुन्छ ?	.... दिन		
82.2b **प्रतिदिन कति समयसम्म गर्नुहुन्छ ?**	..........घण्टा......... मिनेट		

**83. Travel time आवत जावत गर्दाको समयमा गर्ने शारीरिक क्रियाकलाप**<br>अब म तपाईलाई प्रायजसो तपाईले **आवत जावत** (*जस्तै काम गर्न जाँदा, किनमेल गर्न जाँदा, बजार जाँदा, पूजा गर्न जाँदा आदि*) कसरी गर्नुहुन्छ , त्यसबारे केहि प्रश्नहरु सोध्न चाहन्छु ।		**Code**
**83.1** तपाईले आवतजावतका लागि कम्तिमा १० मिनेट हिंड्नु वा साइकलको प्रयोग गर्नुहुन्छ?	**1.** गर्छु , भने **83.2** मा जाने<br>**2.** गर्दिन , भने **84** मा जाने	Bp3.1
**83.2** यदि गर्छु भने, हप्तामा कति दिन ?	.... दिन	Bp3.2
**83.2** तपाईले दिनमा कति समय हिंडडुल वा साइकल चलाएर बिताउनुहुन्छ ?	.... घण्टा....................... मिनेट	Bp3.3

**क्र.सं.**	**प्रश्नहरु**	**उत्तर**	**कोड**
**84.**	प्राय दिनैजसो कति समय तपाई आराम गरेर बिताउनुहुन्छ ? (जस्तै : पढेर, बुनाई गर्दै, टेलिभिजन हेरेर आदि)	..........घण्टा......... मिनेट प्रति दिन	Bp4

अब म तपाईका बालबालिकाहरु (बच्चाहरु)को शारीरिक क्रियाकलापको बारेमा सोध्नेछु ।

यदि सहभागी (उत्तरदाता)को एकभन्दा बढी १ देखि ७ वर्षका बालबालिका छन् भने प्रत्येकलाई बेग्लाबेग्लै नम्बर दिई राख्ने जस्तै १, २, ३ आदि (बढीमा ३ जनाको मात्र) र प्रत्येक बच्चाको जानकारी वा उसकै नाम भएको छेउमा लेख्ने । प्रत्येक प्रश्नको उत्तरमा प्रत्येक बच्चाका लागि उही कोड नं. को प्रयोग गर्ने । यदि एउटा मात्र बच्चा भए बच्चा १ मात्र लेख्ने

**क्र.सं.**	**प्रश्नहरु**	**उत्तर**	**कोड**
**85.**	तपाईको बच्चा विद्यालय जान्छ ?	(see table below)	Bp4.1<br>Bp4.2<br>Bp4.3

**प्रश्न**	**बच्चा १**	**बच्चा २**	**बच्चा ३**
जान्छ			
जाँदैन भने, (प्रश्न नं. **89** मा जाने)			

**क्र.सं.**	**प्रश्नहरु**	**उत्तर**	**कोड**
**86.**	तपाईको बच्चा सँधैजसो कसरी (वा कुन साधनमा) विद्यालय जाने गर्छ ?<br>*(दायाँतिर भएका उत्तरहरु तुरुन्तै नभन्नुस् । प्रश्न सोधेपछि केही समय पर्खनुस् । यदि सहभागीलाई उत्तर भन्न गाह्रो भएमा मात्र उक्त उत्तरहरु पढ्नुहोला ।)*	(see table below)	Bp5.1<br>Bp5.2<br>Bp5.3

**प्रश्न**	**बच्चा १**	**बच्चा २**	**बच्चा ३**
1. पैदल (हिंड्दै)			
2. विद्यालयको बसमा			
3. कार वा मोटरसाइकलमा			
4. साइकलमा			
5. अन्य (उल्लेख गर्ने) ____________			
6. थाहा छैन वा भन्न सक्दिन			

<table>
<tr><td>87.</td><td>तपाईको बच्चा विद्यालय आउँदा-जाँदा कति समय हिंड्ने गर्छ?(बसमा जाने भए, बस चढ्ने ठाउँसम्मको समय)</td><td>बच्चा १ ....घण्टा........ मिनेट प्रतिदिन
बच्चा २ ....घण्टा........ मिनेट प्रतिदिन
बच्चा ३ ....घण्टा........ मिनेट प्रतिदिन</td><td>Bp6.1
Bp6.2
Bp6.3</td></tr>
<tr><td>88.</td><td>तपाईको बच्चाले दिनमा कति समय विद्यालयको गृहकार्य गरेर बिताउँछ ?</td><td>बच्चा १ ....घण्टा........ मिनेट प्रतिदिन
बच्चा २ ....घण्टा........ मिनेट प्रतिदिन
बच्चा ३ ....घण्टा........ मिनेट प्रतिदिन</td><td>Bp7.1
Bp7.2
Bp7.3</td></tr>
<tr><td>89.</td><td>तपाईको बच्चा खाली समय वा फुर्सदको समय (गृहकार्य सकेपछि वा विदाको समय) कसरी बिताउँछ ?

(बहु-उत्तर प्रश्न)
(Multiple answer question)
(दायाँतिर भएका उत्तरहरु तुरुन्तै नभन्नुस् । प्रश्न सोधेपछि केही समय पर्खनुस् । यदि सहभागीलाई उत्तर भन्न गाह्रो भएमा मात्र उक्त उत्तरहरु पढ्नुहोला ।)</td><td>
<table>
<tr><th>प्रश्न</th><th>बच्चा १</th><th>बच्चा २</th><th>बच्चा ३</th></tr>
<tr><td>1. पुस्तकहरु पढेर</td><td></td><td></td><td></td></tr>
<tr><td>2. घरमा एक्लै खेलेर</td><td></td><td></td><td></td></tr>
<tr><td>3. घरमा साथीहरुसँग खेलेर</td><td></td><td></td><td></td></tr>
<tr><td>4. घरबाहिर खेलेर</td><td></td><td></td><td></td></tr>
<tr><td>5. टेलिभिजन हेरेर, कम्प्युटर वा मोबाइल फोनमा (गेम) खेलेर</td><td></td><td></td><td></td></tr>
<tr><td>6. अन्य (उल्लेख गर्ने)
______________________</td><td></td><td></td><td></td></tr>
<tr><td>7. थाहा छैन वा भन्न सक्दिन</td><td></td><td></td><td></td></tr>
</table>
</td><td><u>Child 1:</u>
Bp8.1.1
Bp8.1.2
Bp8.1.3
Bp8.1.4
Bp8.1.5
Bp8.1.6
Bp8.1.7

(For child 2and 3 – similar format: Bp8.2.1 etc)</td></tr>
<tr><td>90.</td><td>तपाईको बच्चा सामान्यतया घरबाहिर हरेक दिन (विदाको दिन बाहेक) कति समय खेल्ने गर्छ (साथी, छिमेकी वा आफन्तको मा समेत) ?</td><td>बच्चा १ ....घण्टा........ मिनेट प्रतिदिन
बच्चा २ ....घण्टा........ मिनेट प्रतिदिन
बच्चा ३ ....घण्टा........ मिनेट प्रतिदिन</td><td>Bp9.1
Bp9.2
Bp9.3</td></tr>
<tr><td>91.</td><td>विदाको दिनमा (शनिबार वा अन्य पर्वमा) तपाईको बच्चा सामान्यतया घरबाहिर कति समय खेल्ने गर्छ (साथी, छिमेकी वा आफन्तको मा समेत) ?</td><td>बच्चा १ ....घण्टा........ मिनेट प्रतिदिन
बच्चा २ ....घण्टा........ मिनेट प्रतिदिन
बच्चा ३ ....घण्टा........ मिनेट प्रतिदिन</td><td>Bp10.1
Bp10.2
Bp10.3</td></tr>
<tr><td>92.</td><td>तपाईको बच्चा सामान्यतया हरेक दिन (विदाको दिन बाहेक)कति समय कम्प्युटर वा मोबाइल फोनमा (गेम) खेल्ने वा टेलिभिजन हेर्ने गर्छ ?</td><td>बच्चा १ ....घण्टा........ मिनेट प्रतिदिन
बच्चा २ ....घण्टा........ मिनेट प्रतिदिन
बच्चा ३ ....घण्टा........ मिनेट प्रतिदिन</td><td>Bp11.1
Bp11.2
Bp11.3</td></tr>
<tr><td>93.</td><td>विदाको दिनमा (शनिबार वा अन्य पर्वमा) तपाईको बच्चा सामान्यतया कति समय कम्प्युटर वा मोबाइल फोनमा (गेम) खेल्ने वा टेलिभिजन हेर्ने गर्छ ?</td><td>बच्चा १ ....घण्टा........ मिनेट प्रतिदिन
बच्चा २ ....घण्टा........ मिनेट प्रतिदिन
बच्चा ३ ....घण्टा........ मिनेट प्रतिदिन</td><td>Bp12.1
Bp12.2
Bp12.3</td></tr>
</table>

VI. आमा र बच्चाको शारीरिक नापतौल

अब म तपाईको र तपाईको बच्चाहरुको उचाई र तौल नाप्ने छु ।			
**94.**	के तपाई अहिले गर्भवती हुनुहुन्छ ?(आमालाई सोध्ने)	1. अँ, छु । (प्रश्न नं. **99** मा जाने)<br>2. अँह, छुइन	Ba1
**95.**	आमाको उचाइ	________________(सेन्टिमिटर)	Ba2
**96.**	आमाको तौल	________________(किलोग्राम)	Ba3
**97.**	आमाको कम्मरको नाप	________________(सेन्टिमिटर)	Ba4
**98.**	आमाको पुट्ठा वा चाकको नाप	________________(सेन्टिमिटर)	Ba5
**99.**	के तपाईलाई उच्च रक्तचापको बिमारी छ ?	**1.** **छ ,** *भने 100 मा जाने* ☐<br>**2.** **छैन ,** *भने 101 मा जाने* ☐	Ba6
**100.**	के तपाई उच्च रक्तचापको कुनै औषधी लिदैं हुनुहुन्छ ?	**1.** अँ , लिदैंछु ☐<br>**2.** अहँ , छैन ☐	Ba7
**101.**	आमाको रक्तचाप	(see table below)<br>**पहिलो, दोस्रो र तेस्रो रक्तचाप लिँदा कम्तीमा ५ मिनेटको फरकमा लिने**	Ba8.1<br>Ba8.2<br>Ba8.3
**102.**	बच्चाको उचाई (सेन्टिमिटरमा)	(see table below)	Ba9.1<br>Ba9.2<br>Ba9.3
**103.**	बच्चाको तौल (किलोग्राममा)	(see table below)	Ba10.1<br>Ba10.2<br>Ba10.3

101. आमाको रक्तचाप:

	**पहिलो रक्तचाप**	**दोस्रो रक्तचाप**	**तेस्रो रक्तचाप**
**SBP**			
**DBP**			

102. बच्चाको उचाई (सेन्टिमिटरमा):

**बच्चा १**	**बच्चा २**	**बच्चा ३**

103. बच्चाको तौल (किलोग्राममा):

**बच्चा १**	**बच्चा २**	**बच्चा ३**

घरमा भएको समन्वय सम्बन्धि बिचार

**बिचार**	**उत्तरदाताको**	**अन्तरवार्ताकारको**
**1.** एकदम राम्रो<br>**2.** ठिकठिकै<br>**3.** गाह्रो अनुभव<br>**4.** उत्तर दिन नचाहेको	☐	☐

तपाईंलाई धन्यवाद ।।।
